# The Effect of Food Value Chain Interventions on Food Security in Sub‐Saharan Africa: A Systematic Review and Meta‐Analysis

**DOI:** 10.1002/fsn3.71881

**Published:** 2026-06-15

**Authors:** Andre M. N. Renzaho, Rashid Abdulai, Kh Shafiur Rahaman, Sanjay Kumar Das, Ramadhani Noor, Stanley Chitekwe

**Affiliations:** ^1^ School of Medicine, Translational Health Research Institute Western Sydney University Penrith New South Wales Australia; ^2^ UNICEF Ethiopia | UNECA Compound Addis Ababa Ethiopia

**Keywords:** dietary diversity, food consumption, food security, food value chain, malnutrition

## Abstract

This study conducted a systematic review and meta‐analysis to examine the quantitative associations between food value chain (FVC) interventions and household food security outcomes in sub‐Saharan Africa. Seven databases with no date restrictions were searched from 1 July 2024 to 25 February 2025. Pooled mean differences (PMDs) with 95% confidence intervals (CIs) were estimated using random‐effects models (*I* ^2^ > 50%). Of 739 studies screened, 31 met the inclusion criteria, including four multi‐country studies spanning 10 countries, yielding 37 study units. Of these, 73% were quasi‐experimental studies, 13.5% were cluster randomized controlled trials, and 13.5% were individually randomized controlled trials. Relative to non‐participants, household participation in FVC interventions was associated with a 16‐percentage‐point lower prevalence of food insecurity (95% CI: −20.13, −11.01; *p* < 0.001), higher food composition scores (FCS; PMD = 6.83%; 95% CI: 1.25, 12.41; *p* = 0.017), greater dietary diversity (DD; PMD = 0.48; 95% CI: 0.23, 0.72; *p* < 0.001), and higher daily energy intake (PMD = 506 kcal; 95% CI: 176, 837; *p* < 0.01). Food expenditure was approximately USD 42 higher per person per year (PMD = 42.33; 95% CI: 21.94, 62.71; *p* < 0.001), and nutrient adequacy scores were higher (PMD = 0.14; 95% CI: 0.04, 0.25; *p* < 0.01). Relative to non‐participants, children living in households participating in FVC interventions had higher weight‐for‐age *z*‐scores (PMD = 0.18; 95% CI: 0.03, 0.34). At the producer level, household participation in FVC interventions was associated with higher farm income per hectare (PMD = USD 45; 95% CI: 19.17, 70.04; *p* < 0.001) and lower post‐harvest losses (PMD = −4%; 95% CI: −8.4, −0.2; *p* < 0.05). No statistically significant associations were observed for height‐for‐age, weight‐for‐height, or maize aflatoxin levels. Overall, household participation in FVC interventions was consistently associated with more favorable household food security outcomes, although findings are predominantly derived from quasi‐experimental evidence. With substantial heterogeneity was observed across several outcomes, particularly for DD and FCS, the findings should be interpreted with caution. Future programmes may benefit from participatory co‐design and closer alignment with local cultural contexts, markets, and food‐system dynamics.

## Graphical abstract

Household participation in food value chain interventions in sub‐Saharan Africa was associated with 16‐percentage‐point lower food insecurity prevalence, higher dietary diversity, 506 kcal greater daily energy intake, USD 42 higher annual food expenditure per person, and improved child weight‐for‐age. Producer‐level benefits included USD 45 higher farm income per hectare and 4% lower post‐harvest losses.

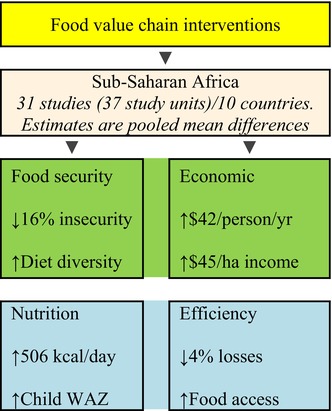

## 1 Introduction

According to the 2024 Revision of the World Population Prospects, Sub‐Saharan Africa (SSA) has experienced the fastest population growth and urbanization globally over the past 63 years (United Nations 2024). The region's population increased more than fivefold, from 217 million in 1960 to 1.20 billion by January 2023. This increase is far greater than the 2.7‐fold global increase (from 2.99 to 8.06 billion) during the same period. With the global population projected to rise by nearly 2 billion by 2050 (United Nations 2024), food demand will pose a major challenge for SSA. Rapid, informal urbanization, a youthful population, and ongoing population growth will strain agricultural and food systems, with broad economic, social, and political implications (OECD, UN ECA, and AfDB 2022).

Global agriculture produces approximately 4 billion tonnes of food annually, enough to feed more people than currently live on Earth and even the projected 9.7 billion by 2050 (Fox and Fimeche 2013). Yet, an estimated 1.2 to 2 billion tonnes (30%–50%) of food is wasted due to inefficiencies in harvesting, storage, transport, and consumption. Such waste results not only in lost food but also in the waste of vital resources used in its production. Kummu et al. (2012) estimated that 25% of global food production, equivalent to 614 kcal per person per day, is lost across the food supply chain. These figures underscore that addressing hunger requires not just increasing production, but improving efficiency, reducing losses, and managing resources more sustainably across the food value chain.

Meeting future food demand amid rapid population growth requires more than increasing production; the priority must shift toward equitable food distribution and strengthened value chain management. SSA is well‐positioned to support these goals. The region holds 60% of the world's remaining uncultivated arable land (Adesina 2018). Of its estimated 400 million hectares of arable land, only 40 million (10%) are currently cultivated (Adesina 2018). Notably, since 2000, SSA has outpaced other low‐ and middle‐income countries in agricultural growth (Odusola 2021). However, this growth has primarily resulted from land expansion and traditional fallowing systems, not increased productivity, farm intensification, or technological adoption (Odusola 2021). Despite its potential to become a global agricultural powerhouse, the region continues to struggle with low productivity and unsustainable development due to underinvestment and systemic barriers. Unlocking this potential will require strategic investments in infrastructure, innovation, and inclusive value chains to convert its vast natural assets into equitable and sustainable food systems.

Harnessing the food value chain (FVC) continuum is essential to addressing growing food demand. The FVC continuum refers to the interconnected agricultural and economic activities spanning pre‐harvest to consumption, aimed at enhancing the quality and market value of food products. However, challenges can arise at any point in this chain. At the production level, land tenure insecurity is a major constraint to FVC interventions (Simbizi et al. 2014). In SSA, where 90% of land is held under customary systems lacking formal legal recognition, overlapping and unclear use rights can hinder investment and long‐term planning (Coyle 2015; Gerken 2023; Holmen 2015; Nelson 2004; Simbizi et al. 2014). This insecurity contributes to land foreignization (Nolte et al. 2016). Beyond land issues, the integration of pre‐ and post‐harvest services, processing, and distribution also faces significant barriers (Adediran et al. 2024; Delgado et al. 2021).

FVC interventions aim to reduce losses, boost revenues, and strengthen consumer demand. Yet, SSA struggles with resource degradation and low productivity characterized by high input costs, poor infrastructure, weak market linkages, inadequate storage, and fragmented regulatory frameworks (De Brauw and Bulte 2021; Dong 2021; Ehui and Pender 2005). Marketing systems lack value‐adding infrastructure such as processing and packaging facilities (Acharya 2003; Nitidae 2022; Purohit et al. 2017). Further barriers include the lack of understanding of the added value of agricultural cooperatives, poorly designed policies focused on finance over functionality, asymmetrical market information, price instability, and weak export incentives (Chitete et al. 2024; Lencucha et al. 2020; Qorri and Felfoeldi 2024). While FVC policies in SSA have evolved from informal to formal mechanisms (de Brauw et al. 2021), the process has been uneven. Inadequate food policies have been characterized by minimal food regulations, poor consumer trust, uncoordinated marketing, neglect of conservation, and weak farmer bargaining power (De Brauw and Bulte 2021; De Janvry and Sadoulet 2005; Ehui and Pender 2005; Webber and Labaste 2009). Only a few countries have frameworks to address unfair trade, and enforcement remains weak (Illy 2012; Schonwetter 2024).

Despite the above challenges, the role of effective FVC policies in addressing food insecurity in SSA remains underexplored and underappreciated. This gap is urgent given that the region allocates only 6% of public spending to food and agriculture, with 20% of this left unspent due to disbursement delays and implementation challenges (Pernechele et al. 2021). As a net food importer, relying on imports for 85% of food needs at an annual cost of USD 48.7 billion (Food and Agriculture Organisation and African Union Commission 2021), the region urgently needs evidence on effective, scalable interventions to boost productivity, reduce import dependence, and enhance food security. To address this gap, the following research question guided the review: what are the quantitative associations between FVC interventions and household food security outcomes in SSA?

## 2 Methods

The preferred reporting items for systematic review and meta‐analysis guidelines (Moher et al. 2010) guided the conduct of this systematic review.

### 2.1 Source of Information

The systematic review was registered with the PROSPERO international prospective registry (CRD42024569988). The search was conducted from 1 July 2024 to 25 February 2025 with no date restrictions. Electronic databases searched included Medline (Ovid), Embase, Web of Science, ProQuest, Cochrane Library, CINAHL, SCOPUS/Elsevier, and Google Scholar (first 200 records screened). Reference lists of included studies were also manually reviewed for additional sources.

### 2.2 Search Strategy

The initial search was conducted by one researcher (KR) with support from a senior librarian, using the concepts: “impact” AND “value chain” (OR “land tenure” OR “agricultural marketing regulation”) AND “food security and nutrition” AND “sub‐Saharan Africa.” Search terms were based on MeSH or subject headings, truncation symbols (*), and Boolean operators. The full extended search strategy is presented in Table S1.

### 2.3 Inclusion

Inclusion criteria were guided by the PICOTS framework (Population, Intervention, Comparison, Outcome, Timing, and Setting) (Riva et al. 2012), as detailed in Table 1. Only experimental and quasi‐experimental studies were included if they focused on interventions enhancing food security along the FVC continuum (Farmery et al. 2021; Food and Agriculture Organisation and Unitens Nations Industrial Development Organization 2024; Graef et al. 2015; Mechri et al. 2023; Nguyen 2018; Wieben 2019). Eligible interventions included (Table 2):

- Value‐adding strategies that harness natural resources such as land, water, soil, and biodiversity.
- Waste management and bioenergy upgrading, including practices such as mulching, animal feed from crop residues, biogas production from food waste, composting, wastewater irrigation, and cooking with food waste.
- Income generation and market strategies, such as savings and credit cooperatives, warehouse receipt systems, guarantee systems, vertical and horizontal coordination, out‐grower schemes, and communication tools.
- Functional upgrading to support actors in moving up the value chain through new roles like bulking, transporting, grading, processing, and marketing.
- Coordination and business model upgrades, aimed at formalizing horizontal (e.g., farmer‐to‐farmer) or vertical (e.g., farmer‐to‐processor/distributor) relationships through contracting.
- Food processing and consumption upgrading, such as food preservation techniques, oil extraction, fortification of staple foods, and improved food storage devices.

**TABLE 1:** The PICOTS framework.

Population	All people of any age or households in any setting. Not restricted by personal or household characteristics
Intervention	Explanatory variables include FVC strategies along with agricultural production, food storage and distribution, food processing and retail/trade, food market regulation, and food consumption
Comparison	A control group in experimental studies (randomized controlled trials‐RCTs) or baseline and/or follow‐up data for quasi‐experimental studies, for example, one‐group pre‐test‐post‐test design, interrupted time series design or repeated cross‐sectional studies; post‐test only design with non‐equivalent groups, untreated control group with pretest and post‐test samples
Outcome	Food security (Doustmohammadian et al. 2022): Food availability: own production or the market, including food stocks, average dietary energy supply adequacy, average value of food production, dietary energy supply from cereals, roots, and tubers, or average of protein supply Food access: household resources and purchasing power, physical and policy environment such as trade, transport infrastructure, cross‐border regulations, per capita gross domestic product in purchasing power equivalent, domestic food price index, the ratio of food expenditure of the poor to total expenditure, food deficit or government subsidies or incentives Food utilization: food consumption, food wastage, undernourishment prevalence, child malnutrition, and micronutrient deficiencies, and Food stability: food import dependency ratio, value of food imports over total exports, volatility in domestic food price, per capita food production variability, per capita food supply variability, coping strategies, and resource conservation.
Timing	The search was carried out from 1 July 2024 to 25 February 2025, with no restriction applied on date
Setting	Sub‐Saharan Africa

**TABLE 2:** Interventions across the FVC continuum: Data extraction criteria matrix.

Stage	Key activities	Main actors	Value addition
1. Input supply	Provision of seeds, fertilizers, tools, irrigation, credit, knowledge Risk mitigation against climate variability and extreme weather events	Agro‐dealers, extension services, seed companies	Quality inputs improve productivity, sustainability, and resilience
2. Production	Diversified farming activities and technologies, enhancing the health of the environment and the adoption of technologically enhanced organic agricultural activities, and enhanced pest and disease management	Farmers, producers, cooperatives	Efficient and diverse production increases yields and food security
3. Processing	Cleaning, sorting, milling, cooking, packaging, preservation	Processors, small industries, food companies	Enhances nutrition quality, shelf‐life, safety, and marketability; supports food loss reduction
4. Storage	Post‐harvest handling, cold storage, warehousing	Producers, distributors, warehouse managers	Prevents spoilage and maintains nutritional value
5. Transportation	Moving food from farms to markets or processors	Transporters, logistics providers	Increases access, reduces delays and losses
6. Distribution	Wholesaling, retailing, food assistance, market access	Traders, retailers, NGOs, government agencies	Enables food availability and affordability across regions
7. Retail and marketing	Selling food in local markets, supermarkets, street vendors, online platforms	Retailers, marketers, small and medium enterprises	Nutrition‐sensitive value chain, ensures consumer access and choice; and creates employment
8. Consumption	Household food preparation and eating practices	Consumers, households	Nutritional outcomes, health impacts, and cultural value
9. Waste management	Food recovery, composting, recycling, food rescue initiatives	Households, businesses, non‐government organizations	Reduces environmental impact and enhances sustainability
10. Governance and policy	Regulations, subsidies, quality standards, trade policies	Governments, donors, regulators, international bodies	Shapes the enabling environment for value chain efficiency and equity
11. Food systems perspective	Interconnected holistic approach and sustainable practices	Agro‐dealers, extension services, seed companies, retailers, marketers, small and medium enterprises, farmers, producers, cooperatives governments, donors, regulators, international bodies, distributors, warehouse managers, households, businesses, non‐government organizations	Integrates all aspects of the FVC, from production to consumption through environmentally, socially, and economically viable options

*Note:* Adapted from (Food and Agriculture Organisation 2021; Food and Agriculture Organisation and Unitens Nations Industrial Development Organization 2024).

Based on the PICOTS framework, potential outcome measures included (Doustmohammadian et al. 2022; Renzaho and Mellor 2010):

1. Overall prevalence of food insecurity

- Definition: Proportion of households reporting either:
  ○ Moderate insecurity: Compromised quality and/or quantity of food consumed, or
  ○ Severe insecurity: Reduced food intake and disrupted eating patterns.

2 Food utilization

- Bio‐utilization of food (nutritional outcomes)

Nutritional status of children based on WHO growth standards.

- Height‐for‐age *Z*‐score (HAZ): Reflects chronic undernutrition.
- Weight‐for‐age *Z*‐score (WAZ): Composite measure of both acute and chronic undernutrition.
- Weight‐for‐height *Z*‐score (WHZ): Indicator of acute malnutrition.
- Body mass index *Z*‐score (BMIz)

Any blood test for vitamin and mineral deficiencies

b Physical utilization (dietary intake measures)

- Food composition score (%): Aggregate measure of household dietary diversity and frequency over the past 7 days.
- Dietary diversity score: Number of different food groups consumed in a specific time period.
- Minimum dietary diversity: Proportion of individuals consuming at least 5 of 8–10 defined food groups in the previous 24 h.
- Nutrient adequacy ratio: Individual intake of 18 micronutrients divided by age‐ and sex‐specific dietary reference intakes.
- Energy intake (kcal/person/day): Daily caloric intake per person.

3 Food availability

- Crop yield equivalent (kg/ha): Quantity of a crop produced per hectare.
- Farm food yield revenue (USD per capita): Value of agricultural output per person, used as a proxy for food purchasing power.
- Agricultural diversity score: Number and variety of crop and livestock types produced by a household or farm.
- Post‐harvest food loss (% or volume): Food lost between harvest and storage (includes losses from transporting, drying, shelling, winnowing).
- Aflatoxin infestation: Presence and levels of aflatoxins in stored cereals at household level.

4 Food access

- Per capita total consumption expenditure (USD/year): Total value of food consumed per individual annually.
- Poverty level or multidimensional poverty index, which is a composite index measuring overlapping deprivations in health, education, and living standards.

5 Food stability

- Coping strategy index: Frequency and severity of coping strategies used when facing food shortages.
- Months of adequate household food provisioning: Number of months in a year when households report sufficient food.
- Food import dependency ratio: Share of imported food in total food supply.
- Value of food imports over total exports (%): Indicator of a country's economic vulnerability to external food supplies.
- Volatility in domestic food prices: Fluctuation in national food prices over time.
- Variability of per capita food production: Fluctuation in food production per person.
- Variability of per capita food supply: Fluctuation in food availability per person.
- Resource conservation practices: Extent of use of sustainable natural resource management strategies.

Exclusion criteria:

Given the varying degree of heterogeneity, poor design, and poor data quality in agricultural interventions (Silva et al. 2024), we employed stringent exclusion criteria.

- Geographical scope: Studies conducted outside SSA were excluded.
- Study type: we focused on
  ○ Predictive modeling studies.
  ○ Archaeological and historical studies
  ○ Non‐comparative descriptive studies
  ○ Review articles, conference abstracts, protocols, and other forms of gray literature.
  ○ Studies with inadequate methodological information.
- Topical Scope:
  ○ Studies focused on plant diseases or plant species, or seasonal variability in species.
  ○ Studies investigating land inheritance, restorative justice, grievance mechanisms, including alternative dispute resolution or law enforcement capacity building.
  ○ Studies monitoring carbon footprints or other environmental assessments not linked to food security outcomes.
- Field product testing: Excluded were:
  ○ Studies that tested agricultural products such as seed varieties, pesticides, fertilizers, storage materials, or bio‐stimulants, for the purpose of establishing efficacy, performance, or safety
- Language:

Only studies published in English were included.

### 2.4 Data Screening and Extraction

All identified studies were exported into EndNote for deduplication. Screening was conducted in three sequential stages: title screening, abstract screening, and full‐text review. Data extraction was guided by the Joanna Briggs Institute data extraction form (Aromataris et al. 2024). To ensure consistency and reduce variability in data collection, a data extraction criteria matrix was developed, informed by current advances in FVC research (Table 2) (Farmery et al. 2021; Food and Agriculture Organisation and Unitens Nations Industrial Development Organization 2024; Food and Agriculture Organization 2017; Graef et al. 2015; Mechri et al. 2023; Nguyen 2018; Wieben 2019). One reviewer (KR) conducted the initial screening and data extraction, while a senior researcher (AR) independently validated the process using a random 40% sample of included studies. Any discrepancies between the two reviewers were resolved through discussion. Where consensus could not be reached, a third reviewer was consulted to adjudicate.

### 2.5 Risk Assessment

Data were analyzed using Stata version 18. Risk of bias was assessed using the Revised Cochrane Risk of Bias Tool for RCTs (Cochrane Collaboration 2019), and non‐randomized studies (Seo et al. 2023). Quality assessment was initially conducted by one researcher (KR). A random subset of 15 studies was independently assessed by a second researcher (AR) to validate the ratings. Inter‐rater reliability was determined using Cohen's kappa coefficient, yielding a value of 0.768, indicating substantial agreement.

### 2.6 Meta‐Analysis Approach

Heterogeneity across studies was assessed using the *I* ^2^ statistic, and random‐effects models were used for all meta‐analyses. Publication bias and small‐study effects were examined using Egger's regression test (Egger et al. 1997), complemented with a visual inspection of funnel plots.

#### 2.6.1 Subgroup Analyses

Subgroup analyses were pre‐specified to identify varying effects by FVC intervention type, study design, and region, thereby minimizing post hoc fishing expeditions and false‐positive findings (Clark et al. 2022). Unadjusted estimates were unavailable across included studies, which remains a recognized challenge in agricultural intervention research. In this field, program effects are commonly estimated using econometric approaches such as regression adjustment, propensity score matching, or switching regression models (Gertler et al. 2016; World Bank Independent Evaluation Group 2011). For consistency, all reported mean differences (MD) ± standard errors (SE) were derived from models adjusted for relevant confounding factors within each study (Voils et al. 2011). This ensured that pooled estimates under the random‐effects model accounted for both between‐study variation and differences in baseline characteristics or covariates that could influence observed effects. While the Cochrane Handbook recommends ≥ 10 studies for subgroup analyses (Cochrane Collaboration 2011; Page et al. 2021), the multidimensional nature of food security presents unique challenges. Indicators assess distinct dimensions (access, availability, utilization, stability) at different scales (individual, household, national) (Jones et al. 2013), making standardization into a single Cohen's *d* inappropriate (Cuijpers 2021; Cummings 2011). Cohen's *d* is highly dependent on the specific measure and population, and heterogeneity across food security outcomes limits its interpretability and comparability (Cuijpers 2021; Cummings 2011). An intervention may have large effects on one measure (e.g., dietary diversity) but minimal effects on another (e.g., food expenditure or child anthropometric outcomes), preventing direct comparisons across studies. Given these constraints, subgroup analyses were conducted with ≥ 6 studies where the ≥ 10‐study threshold could not be met, and this limitation is explicitly acknowledged.

#### 2.6.2 Mean Difference Calculation

Mean differences (MDs) were calculated as pooled mean differences (PMDs) with 95% confidence intervals (CIs). Meta‐analyses included single‐study estimates where evidence was scarce (Rojas‐Reyes and Orrego‐Rojas 2015). To ensure consistency, the direction of effect was set a priori for all outcomes (MD = treatment group mean − comparator group mean). For difference‐in‐differences (DiD) analyses, the estimate represented the mean difference in change (pre‐ to post‐intervention) between treatment and comparator groups as follows:

$$\mathrm{DiD}=\left({\text{Mean}}_{t,\text{post}}-{\text{Mean}}_{t,\mathrm{pre}}\right)-\left({\text{Mean}}_{c,\text{post}}-{\text{Mean}}_{c,\mathrm{pre}}\right)$$

where (Mean_t,post_‐Mean_t,pre_) represents MD in the treatment, that is, the change over time in the treatment group and (Mean_c,post_ − Mean_c,pre_) represents MD in the control, that is, the change over time in the control group.

This approach accounts for temporal trends and baseline differences, providing more robust estimates of intervention effects (Lin et al. 2025; Zhou et al. 2016). Negative Mean differences indicated reductions in outcome measures (intervention group < comparator group), while positive values indicated increases.

#### 2.6.3 Data Handling

For factorial designs with multiple intervention arms, outcome data were combined across relevant arms. Multi‐country studies were analyzed as separate studies for each country (Pan et al. 2018). DiD estimates incorporated into multivariate regression models (controlling for observed covariates) were extracted when available, as these models handle staggered intervention adoption and improve precision. However, studies varied considerably in data quality and design (Silva et al. 2024), and some did not provide parameters required for meta‐analysis. When standard errors or standard deviations were missing or incorrectly reported, they were recalculated using the RevMan calculator tool. For studies employing multiple analytic models (e.g., propensity score matching [PSM], inverse probability weighting, endogenous switching regression), only results from balanced PSM models were included to ensure consistency and reduce Type I and Type II errors. Among PSM techniques, Nearest Neighbor matching was selected due to its high average accuracy (86.3%) and robustness as a non‐parametric method less sensitive to parametric assumptions (Deng et al. 2023). Outcomes reported in only a single study were not pooled quantitatively, as a single study does not permit estimation of between‐study variability or a meaningful summary effect. In such cases, narrative synthesis was undertaken to describe the direction, magnitude, and context of the findings.

## 3 Results

**FIGURE 1:**
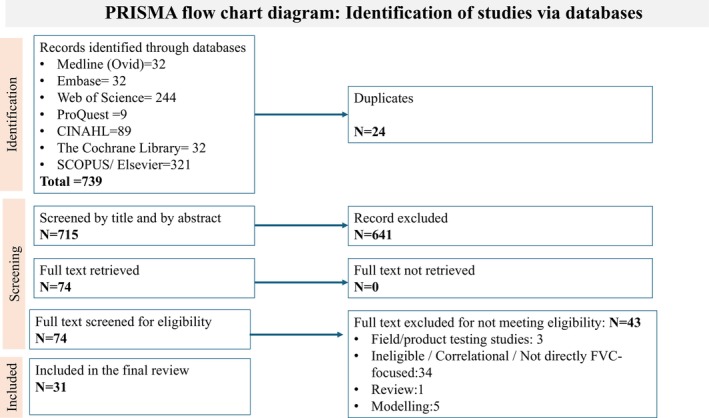
PRISMA flow chart diagram: Identification of studies via databases.

The initial search yielded 739 records, of which 24 duplicates were removed. A total of 715 studies were screened by title and abstract, resulting in the exclusion of 641 studies that did not meet the inclusion criteria. The full texts of 74 studies were assessed for eligibility. Of these, 43 studies were excluded for the following reasons (Table S2): (1) 3 field/product testing studies, (2) 34 studies were ineligible, non‐FVC focused or descriptive, (3) 1 study was a narrative review, and (4) 5 studies were predictive modeling studies. Ultimately, 31 studies met the inclusion criteria and were retained for meta‐analysis (Figure 1). Table 3 provides characteristics of included interventions.

**TABLE 3:** Characteristics of included interventions.

Author	Year	Country	FVC stage	Value addition	Intervention description	Study design	Outcome measure
Abebaw et al.	2010	Ethiopia	Food system approach	Sustainable practices	Integrated program (rehabilitation, irrigation, livestock, roads, crops, vegetables)	QE	Calorie intake
Akinola & Sofoluwe	2012	Nigeria	Production	Food availability	Mulching to improve yam yield and soil health	QE	Yield (kg/ha)
Ali et al.	2022	Ethiopia	Production	Food availability	CSA practices: irrigation, diversification, soil fertility, conservation agriculture	QE	Food security, dietary diversity
Asfaw et al.	2012	Ethiopia	Production	Food availability	Improved chickpea technology	QE	Consumption expenditure/adult
Asfaw et al.	2012	Tanzania	Production	Food availability	Improved pigeonpea technology	QE	Consumption expenditure/adult
Aweke et al.	2021	Ethiopia	Production	Food availability	Improved crop/livestock tech (sorghum, maize, poultry)	QE	Food comp., dietary diversity
Belissa	2024	Ethiopia	Production	Input supply	Weather‐index insurance	QE	Food consumption, ag. input investment
Depenbusch et al.	2021	Kenya	Production	Food availability	Home gardens + seed kits	RCT	Veg. consumption, dietary diversity
Depenbusch et al.	2021	Tanzania	Production	Food availability	Home gardens + seed kits	RCT	Veg. consumption, dietary diversity
Depenbusch et al.	2021	Uganda	Production	Food availability	Home gardens + seed kits	RCT	Veg. consumption, dietary diversity
Hlatshwayo et al.	2023	South Africa	Storage & Distribution	Food access	Market participation by smallholders	QE	Food comp., dietary diversity
Khonje et al.	2020	Zambia	Retail & Consumption	Food utilization	Exposure to modern food retailers	QE	Anthropometry, food variety, diversity
Madsen et al.	2021	Malawi	Production	Food availability	Agroecological practices (crop diversity, legume management)	QE	Food insecurity, dietary diversity
Mango et al.	2017	Zimbabwe	Production	Food availability	Conservation agriculture: minimal tillage, mulching, rotation	QE	Food consumption score
Mango et al.	2017	Mozambique	Production	Food availability	Conservation agriculture: minimal tillage, mulching, rotation	QE	Food consumption score
Mango et al.	2017	Malawi	Production	Food availability	Conservation agriculture: minimal tillage, mulching, rotation	QE	Food consumption score
Mango et al.	2018	Southern Africa	Retail & Consumption	Food access	Small‐scale irrigation	QE	Agricultural income
Olney et al.	2015	Burkina Faso	Retail & Consumption	Food utilization	Homestead food production + nutrition BCC	CRCT	Anthropometry, dietary diversity
Santoso et al.	2021	Tanzania	Retail & Consumption	Food utilization	Agroecology + legume seeds, mentor farmers, nutrition integration	CRCT	Anthropometry, food insecurity, diversity
Maredia et al.	2023	Kenya	Retail & Consumption	Food utilization	Direct marketing of nutritious food (peanut butter)	RCT	Consumption, purchasing
Mekonnen et al.	2022	Ethiopia	Production	Food availability	Small‐scale irrigation (pumps, kits, soil monitoring)	QE	Dietary diversity, anthropometry
Mekonnen et al.	2022	Tanzania	Production	Food availability	Small‐scale irrigation (pumps, kits, soil monitoring)	QE	Dietary diversity, anthropometry
Mkhize et al.	2022	South Africa	Food system approach	Sustainable practices	Legume varieties, nutrition‐sensitive training	QE	Yield, legume consumption
Oduniyi & Chagwiza	2022	South Africa	Food system approach	Sustainable practices	Sustainable land management (mulching, agroforestry, zero tillage)	QE	Yield (kg/ha), food security
Oldewage‐Theron & Egal	2015	South Africa	Production	Food consumption	Nutrition education + soy/veg gardens, recipe training	QE	Dietary diversity, food intake
Pailler et al.	2015	Tanzania	Food system approach	Sustainable practices	Community‐based natural resource management (CBFM, JFM, WMAs)	QE	Food security, wealth, anthropometry
Passarelli et al.	2020	Ethiopia	Production	Food utilization	Chicken production + BCC + home gardening	CRCT	Food expenditure, dietary diversity, anthropometry
Sisay	2023	Ethiopia	Production	Food access	Organic and inorganic soil fertility practices	QE	Net crop revenue, food comp. score
Sissoko et al.	2021	Mali	Production	Food access	Seed priming + fertilizer microdosing	QE	Cereal yield, net value, household expenditure
Wang et al.	2024	Tanzania	Production	Food utilization	School‐based nutrition (meals, gardens, education)	CRCT	Diet quality, food security, anthropometry
Chegere et al.	2020	Tanzania	Storage & Distribution	Food access	Post‐harvest training + hermetic bags	QE	Food insecurity, post‐harvest loss
Gebre et al.	2023	Ethiopia	Retail & Consumption	Food access	On‐farm + off‐farm income diversification	QE	Household food insecurity access scale
Kansanga et al.	2021	Malawi	Production	Food utilization	Agroecology (diversification, legumes, composting, integration)	QE	Production diversity, dietary diversity
Leavens et al.	2021	Senegal	Storage & Distribution	Food access	Post‐harvest safety (training, drying tarps, PICS bags)	CRCT	Aflatoxin exposure
Nkegbe & Mumin	2022	Ghana	Storage & Distribution	Food access	Community markets and storage + rural electrification	QE	Food comp. score, nutrient intake
Omotilewa et al.	2018	Uganda	Storage & Distribution	Food access	Hermetic maize storage (PICS3)	RCT	Storage duration, post‐harvest loss (%)
Zaccharia et al.	2024	Tanzania	Storage & Distribution	Food availability	Hermetic storage technologies (HSTs)	QE	Stored maize (kg), HFIAS

A total of 31 studies met the inclusion criteria, of which four were multi‐country studies spanning 10 countries. Specifically, Depenbusch et al. (2021) reported data from Kenya, Tanzania, and Uganda. Mango et al. (2017) included data from Malawi, Mozambique, and Zimbabwe. Both Asfaw et al. (2012) and Mekonnen et al. (2022) provided data from Tanzania and Ethiopia. Since each country‐specific dataset represented a distinct study context, each was treated as a separate analytical unit, increasing the total number of study units included in the meta‐analysis from 31 to 37. Of these 37 study units, 73% were quasi‐experimental (QE) studies, 13.5% were cluster RCTs (cRCTs), and 13.5% were individual RCTs.

### 3.1 Risk of Bias

Table 4 presents the risk of bias assessment for the included studies. For randomized controlled trials (RCTs), the main threats were performance bias (e.g., most RCTs could not mask participants after allocation or blind staff and personnel delivering the intervention) and detection bias (e.g., there were widespread challenges in blinding outcome assessors). We find that, in agricultural interventions, exposure bias is particularly problematic due to the nature of the interventions and the social dynamics among farmers. This includes (1) allocation concealment issues (i.e., difficulty in masking households or individuals at baseline), (2) the lack of blinding (i.e., inability to blind staff, personnel, and outcome assessors), (3) contamination bias (i.e., challenges in controlling for cross‐intervention exposure or alternative exposure pathways), (4) compliance bias (i.e., inconsistent monitoring of farmer behaviors and adherence to intervention protocols), and (5) co‐investigation bias (i.e., the influence of multiple sources of information or peer learning among farmers). These biases can occur at various stages of intervention delivery and may lead to skewed outcomes or spurious conclusions. Despite these limitations, RCTs showed low risk of selection, attrition, and selective reporting biases, with most implementing proper randomization (see Table 4, Panel A). As shown in Table 4, Panel B, only 42.9% of quasi‐experimental studies used appropriate matching techniques to test for covariate balance between treatment and control groups, which is critical to address selection bias. Notably, none of the quasi‐experimental studies implemented blinding of outcome assessors, indicating a universal risk of detection bias in this study design group.

**TABLE 4:** Risk of bias items across all included studies.

Panel A: Experimental studies			
Criteria	Low	High	Unclear
Randomization sequence generation	8	0	2
Allocation concealment	3	7	0
Blinding staff and personnel (performance bias)	0	10	0
Blinding of outcome assessment (detection bias)	0	9	1
Incomplete outcome data (attrition bias)	9	1	0
Selective outcome reporting	10	0	0
Inappropriate handling of incomplete data	7	3	0

Panel B: Quasi‐experimental studies			
Criteria	Low	High	Unclear
Comparability of the target group (selection bias‐inappropriate comparison)	9	12	0
Target group selection (selection bias‐inappropriate intervention's exposure)	16	4	1
Confounders' selection (selection bias‐inappropriate confounder's consideration)	17	4	0
Measurement of intervention/exposure (performance bias)	19	2	0
Blinding of assessors (detection bias due to inappropriate binding)	0	20	1
Outcome assessment (detection bias‐inappropriate assessment methods)	21	0	0
Incomplete outcome data (attrition bias‐inappropriate handling of incomplete data)	12	7	2
Selective outcome reporting: Reporting bias‐selective outcome reporting	21	0	0

### 3.2 Impact of FVC Intervention on Food Security

**FIGURE 2:**
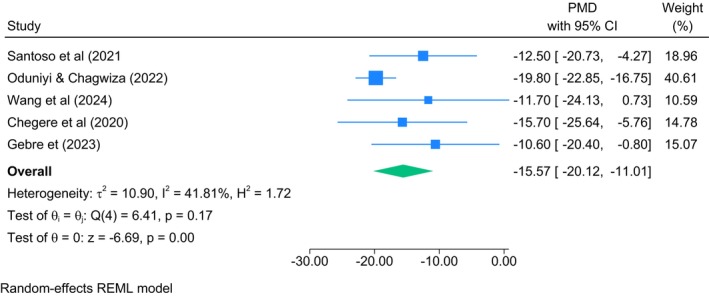
FVC interventions' impact on food insecurity.

Of the 37 included studies, five (*N* = 2135 participants) reported quantitative data on the relationship between FVC interventions and the prevalence of food insecurity as shown in Figure 2 (Chegere et al. 2020; Gebre et al. 2023; Oduniyi and Chagwiza 2022; Santoso et al. 2021; Wang et al. 2024). Overall, households engaged in FVC interventions had a 16‐percentage‐point lower prevalence of food insecurity compared with non‐participants (PMD: −15.57; 95% CI: −20.13 to −11.01; *p* < 0.001). Egger's regression test indicated a significant small‐study effect (*z* = 2.36, *p* < 0.05); however, given the limited number of studies (*n* = 5), this result should be interpreted with caution. The test is underpowered with so few studies, and while it may suggest potential publication bias, the finding is not definitive. Visual inspection of the funnel plot was consistent with this observation, but the small sample limits the reliability of both the test and the plot. Subgroup analyses were not performed due to the insufficient number of studies, limiting the ability to explore differences across study design, FVC intervention type and region.

### 3.3 Impact of the FVC Interventions on Anthropometric Outcomes

Among the 37 studies included in the review, eight studies (*N* = 13,441) reported data on children's HAZ (Khonje et al. 2020; Mekonnen et al. 2022; Olney et al. 2015; Pailler et al. 2015; Passarelli et al. 2020; Santoso et al. 2021; Wang et al. 2024), six studies (*N* = 12,076) reported WHZ, and three studies (*N* = 9518) reported WAZ (Olney et al. 2015; Pailler et al. 2015; Passarelli et al. 2020). As shown in Table 5, Figure S1, children from households engaged in FVC interventions had slightly higher WAZ compared with non‐participants (PMD = 0.18; 95% CI: 0.03–0.34; *p* < 0.05). No consistent associations were observed for HAZ or WHZ, although subgroup analyses suggested a small association for HAZ in quasi‐experimental studies but not in CRCTs, with no differences by region or FVC intervention type. Two studies (*N* = 1365 adolescents) reported BMI‐for‐age *z*‐scores (Khonje et al. 2020; Wang et al. 2024) which were slightly higher among participants compared with non‐participants (PMD = 0.22; 95% CI: 0.02–0.43; *p* < 0.05). Egger's tests did not indicate small‐study effects for HAZ (*z* = 0.47, *p* = 0.64), WAZ (*z* = 1.47, *p* = 0.14), or WHZ (*z* = 2.48, *p* = 0.95). However, the small number of studies for each outcome, coupled with variation in study design and risk of bias, limits confidence in these findings and precludes definitive conclusions regarding the presence or absence of small‐study influences.

**TABLE 5:** Impact of FVC management on anthropometric outcomes among children.

Authors	MD	95% CI		% weight
HAZ (*I* ^2^ = 33.7, *z*‐statistic for theta = 1.84, *p* = 0.066)				
Khonje et al. (2020)	0.210	−0.023	0.443	8.760
Olney et al. (2015)	0.060	−0.070	0.190	19.490
Santoso et al. (2021)	0.078	−0.014	0.170	26.930
Mekonnen et al. (2022)	−0.322	−1.206	0.562	0.750
Mekonnen et al. (2022)	−0.108	−0.925	0.709	0.870
Pailler et al. (2015)	0.228	−0.019	0.475	7.980
Passarelli et al. (2020)	0.200	−0.055	0.455	7.580
Wang et al. (2024)	−0.031	−0.120	0.058	27.630
Weighted PMD	0.073	−0.005	0.150	100
WAZ (*I* ^2^ = 0.00, *z*‐statistic for theta = 2.28, *p* = 0.022)				
Olney et al. (2015)	0.190	−0.234	0.614	13.890
Pailler et al. (2015)	0.220	−0.027	0.467	40.950
Passarelli et al. (2020)	0.150	−0.085	0.385	45.150
Weighted PMD	0.184	0.026	0.342	100
WHZ (*I* ^2^ = 22.43, *z*‐statistic for theta = 1.07, *p* = 0.285)				
Olney et al. (2015)	0.100	−0.390	0.590	4.300
Santoso et al. (2021)	−0.042	−0.164	0.080	38.040
Mekonnen et al. (2022)	0.873	−0.005	1.751	1.390
Mekonnen et al. (2022)	0.203	−0.608	1.014	1.620
Pailler et al. (2015)	0.092	−0.012	0.196	44.160
Passarelli et al. (2020)	0.120	−0.180	0.420	10.500
Weighted PMD	0.057	−0.047	0.161	100

### 3.4 Impact of FVC Interventions on Food Composition and Dietary Diversity Scores

A total of nine studies (*N* = 19,678) reported data on Food Consumption Scores (FCS) (Ali et al. 2022; Aweke et al. 2021; Mango et al. 2017; Nkegbe and Mumin 2022; Oldewage‐Theron and Egal 2015; Sisay 2023; Wang et al. 2024), 13 studies (*N* = 8113) on Household Dietary Diversity Scores (HDDS) (Ali et al. 2022; Aweke et al. 2021; Depenbusch et al. 2021; Hlatshwayo et al. 2023; Kansanga et al. 2021; Khonje et al. 2020; Madsen et al. 2021; Mekonnen et al. 2022; Oldewage‐Theron and Egal 2015; Santoso et al. 2021), and five studies (*N* = 2758) on Individual Dietary Diversity Scores (IDDS) (Depenbusch et al. 2021; Khonje et al. 2020; Passarelli et al. 2020).

As shown in Table 6, Figures S2 and S3, households engaged in FVC interventions were associated with higher FCS (PMD = 6.83; 95% CI: 1.25–12.41; *p* = 0.017) and greater dietary diversity as measured by HDDS (PMD = 0.48; 95% CI: 0.23–0.72; *p* < 0.001) and IDDS (PMD = 0.56; 95% CI: 0.13–1.00; *p* = 0.011), compared with non‐participants. Egger's tests did not indicate small‐study effects for FCS (*z* = 1.75, *p* = 0.08) or HDDS (*z* = 1.37, *p* = 0.17) but suggested potential small‐study effects for IDDS (*z* = −2.72, *p* = 0.007). Given the small number of IDDS studies and high levels of heterogeneity, the intervention's impact may not be consistent across studies. Findings should therefore be interpreted with caution. Subgroup analyses indicated that the association between FVC participation and HDDS was observed across African regions (Figure S3.1), in both CRCTs and quasi‐experimental designs, but not in individually RCTs (Figure S3.2). Associations were also observed across all stages of the food value chain (Figure S3.3). In addition, Mekonnen et al. (2022) reported Women's Dietary Diversity Scores (WDDS) in Ethiopia and Tanzania; however, no overall association with FVC participation was observed (PMD = 0.21; 95% CI: −0.08 to 0.50; *p* = 0.16).

**TABLE 6:** Impact of FVC interventions on food composition and dietary diversity.

Authors	MD	95% CI		% weight
*FCS* (*I* ^2^ = 99.74, *z*‐statistic for theta = 2.4, *p* = 0.017)				
Ali et al. (2022)	56.48	13.80	99.16	1.52
Aweke et al. (2021)	13.53	4.71	22.35	10.09
Mango et al. (2017)	−2.05	−6.13	2.03	12.58
Mango et al. (2017)	5.49	−0.85	11.82	11.49
Mango et al. (2017)	−0.03	−5.97	5.91	11.70
Oldewage‐Theron and Egal (2015)	5.54	1.31	9.78	12.52
Sisay (2023)	3.81	1.33	6.29	13.14
Wang et al. (2024)	1.79	1.65	1.94	13.49
Nkegbe and Mumin (2022)	20.78	20.41	21.15	13.48
Weighted PMD	6.83	1.25	12.41	100.01
*HDDS* (*I* ^2^ = 93.71, *z*‐statistic for theta = 3.83, *p* ≤ 0.001)				
Ali et al. (2022)	3.07	0.75	5.39	1.01
Aweke et al. (2021)	1.22	0.36	2.08	4.77
Depenbusch et al. (2021)	0.17	−0.52	0.86	6.10
Depenbusch et al. (2021)	−0.17	−0.56	0.22	9.03
Depenbusch et al. (2021)	0.22	−0.74	1.18	4.17
Hlatshwayo et al. (2023)	0.77	0.27	1.27	7.88
Khonje et al. (2020)	1.04	0.96	1.12	11.63
Madsen et al. (2021)	0.67	0.16	1.18	7.80
Santoso et al. (2021)	0.57	0.30	0.84	10.31
Mekonnen et al. (2022)	0.11	−0.18	0.41	10.08
Mekonnen et al. (2022)	0.17	−0.36	0.71	7.52
Oldewage‐Theron and Egal (2015)	0.58	0.08	1.07	7.95
Kansanga et al. (2021)	0.18	0.14	0.21	11.75
Weighted PMD	0.48	0.23	0.72	100.00
*IDDS* (*I* ^2^ = 79.27, *z*‐statistic for theta = 2.55, *p* = 0.011)				
Depenbusch et al. (2021)	0.65	−0.04	1.34	17.37
Depenbusch et al. (2021)	−0.09	−0.68	0.50	19.65
Depenbusch et al. (2021)	0.00	−1.45	1.45	6.90
Khonje et al. (2020)	1.01	0.93	1.10	30.53
Passarelli et al. (2020)	0.62	0.27	0.98	25.55
Weighted PMD	0.56	0.13	1.00	100.00
*PCFCE in USD per year* (*I* ^2^ = 0.0, *z*‐statistic for theta = 4.07, *p* ≤ 0.001)				
Asfaw et al. (2012)	50.00	20.60	79.40	48.1
Asfaw et al. (2012)	20.00	−30.96	70.96	16.0
Belissa (2024)	42.00	8.00	76.01	35.9
*Weighted PMD*	*42.33*	*21.94*	*62.71*	100
*Agricultural return (USD/ha)* (*I* ^2^ = 80.34, *z*‐statistic for theta = 3.44, *p* < 0.001)				
Akinola and Sofoluwe (2012)	20.40	5.43	35.37	18.3
Asfaw et al. (2012)	290.00	105.76	474.24	1.74
Asfaw et al. (2012)	120.00	29.84	210.16	5.65
Depenbusch et al. (2021)	58.80	−7.84	125.44	8.34
Depenbusch et al. (2021)	0.70	−13.41	14.81	18.43
Depenbusch et al. (2021)	10.60	−87.40	108.60	5.01
Mango et al. (2018)	35.19	−9.25	79.63	12.24
Sisay (2023)	73.35	31.25	115.46	12.72
Sissoko et al. (2022)	56.01	36.78	75.23	17.57
Weighted PMD	44.60	19.17	70.04	100.00

Few studies reported other dietary indicators, limiting further subgroup analyses. Two studies reported data on energy intake (Abebaw et al. 2010; Khonje et al. 2020), with findings suggesting that FVC participation was associated with higher daily energy intake (PMD = 506 kcal; 95% CI: 176–837; *p* < 0.01). However, Khonje et al. (2020) found that modern retailers contributed to higher consumption of ultra‐processed foods and calories, with the fully adjusted model suggesting that one percentage point increase in the modern retailer expenditure raises caloric intake by 3.6 kcal (or 355 kcal per 100 percentage point increase in the modern retailer expenditure). Additionally, Abebaw et al. (2010) found that integrated food security program raised physical food calorie intake by 30% among the beneficiary households. Although a random‐effects model is acceptable with two studies, between‐study heterogeneity cannot be estimated reliably at this sample size, and the pooled estimate should therefore be interpreted with caution. One study reported data on nutrient adequacy (Oldewage‐Theron and Egal 2015), evaluating whether an integrated food and nutrition intervention in South Africa improved nutrient adequacy. The intervention encompassed home gardening, nutrition education, and recipe development. FVC participation was associated with improved nutrient adequacy; however, as findings are derived from unadjusted analyses, they warrant careful interpretation. One study reported data on the proportion of women and children meeting minimum dietary diversity in rural Tanzania (Santoso et al. 2021), finding that FVC participation was associated with a higher likelihood of meeting minimum dietary diversity in both groups. This study was a cRCT with a low risk of bias. Overall, findings were characterized by substantial heterogeneity, small sample sizes (except for FCS and HDDS), variable study quality, and a predominance of single‐study outcomes. The overall evidence therefore remains inconclusive and cannot be generalized beyond the specific study contexts. The very high heterogeneity observed in the Eastern Africa subgroup likely reflects differences in intervention types, study design, and implementation contexts (Supporting Information, Figure 2).

### 3.5 Food Availability

Nine studies (*N* = 3263) of the 37 included in the review reported agricultural returns measured in USD per hectare (Akinola and Sofoluwe 2012; Asfaw et al. 2012; Depenbusch et al. 2021; Mango et al. 2018; Sisay 2023; Sissoko et al. 2022). As shown in Table 6, Figure S4, households participating in FVC interventions recorded higher agricultural returns, earning on average USD 45 more per hectare (95% CI: 19.17–70.04; *p* < 0.001) and lower post‐harvest losses (PMD = −4; 95% CI: −8.4 to −0.2; *p* < 0.05), compared with non‐participants. Substantial heterogeneity was observed across studies, reflecting variability in effect sizes across study designs and contexts. Egger's regression test additionally indicated evidence of small‐study effects (*z* = 2.76, *p* < 0.01), further suggesting that these associations should be interpreted with caution. Subgroup analyses indicated that the association with higher returns was observed in Eastern and Western Africa, but not in Southern Africa, and was evident in quasi‐experimental studies but not in individual RCTs.

Except for cereals, evidence on food production outcomes was derived from single studies and could not be pooled quantitatively. Three studies reported data on cereal production (Oduniyi and Chagwiza 2022; Omotilewa et al. 2018; Sissoko et al. 2022) with no significant difference observed between adopters and non‐adopters (PMD = 384 kg/ha; 95% CI: −302 to 1069; *p* = 0.27). The small number of studies precluded meaningful assessment of publication bias using Egger's test and limited the precision, interpretability, and generalisability of findings.

One study conducted in north‐west Durban, South Africa evaluated whether the introduction of grain legume varieties for crop diversification improves sustainable food production (Mkhize et al. 2022). Legume yield decreased by 27 kg/ha from Year 1 to 2 (95% CI: −68 to 13), though this was not statistically significant. As findings were based solely on bivariate analyses from a one‐group before‐and‐after design with no comparison group, they represent unadjusted estimates and should be interpreted accordingly.

Zacharia et al. (2024) examined the impact of hermetic storage technologies on maize storage in Tanzania, finding that adoption at the household level increased maize storage quantities by 40% (468 kg; 95% CI: 450–486). However, the study relied on self‐reported data and focused on maize producers' organizations, which typically have better access to input loans and markets. Such factors may independently influence technology adoption and introduce selection bias.

Akinola and Sofoluwe (2012) examined the impact of mulching technology adoption among yam farmers in Osun State, Nigeria. Nearest‐neighbor propensity score matching indicated that adoption was associated with substantially higher yam production per hectare (PMD = +5321 kg; 95% CI: 3754–6887). The robustness of these findings was supported by benchmarking the PSM model through comparison of adopters and non‐adopters on observed pre‐matching characteristics, confirming adequate covariate balance. Leavens et al. (2021) conducted an ex‐post evaluation among 2000 smallholder farming households in Senegal, 2 years after participation in an RCT targeting aflatoxin reduction in stored maize. No clear associations were observed for aflatoxin contamination, either in mean concentration or in the proportion of samples exceeding the 20 parts per billion safety thresholds. Overall, the evidence on food availability, particularly in relation to agricultural returns and production, remains suggestive rather than conclusive, given the limited number of studies and their methodological heterogeneity.

### 3.6 Food Access

Table 6 shows that three studies with 2456 participants (Asfaw et al. 2012; Belissa 2024) reported on food expenditure per person per year. Households participating in FVC interventions spent approximately USD 42 more per person annually (PMD = 42.33; 95% CI: 21.94–62.71; *p* < 0.001). In Kenya, one RCT found a significant increase in household food spending (Maredia et al. 2024). FVC participation was also associated with reduced poverty in Ethiopia (Ali et al. 2022), with the robustness of findings supported by benchmarking the PSM model through comparison of adopters and non‐adopters on observed pre‐matching characteristics, confirming adequate covariate balance. The small number of studies for several outcomes precluded meaningful assessment of publication bias using Egger's test, limiting the precision, interpretability, and generalisability of findings.

## 4 Discussion

This study aimed to quantitatively assess associations between FVC interventions and household food security outcomes in sub‐Saharan Africa. Although the small number of studies and variation in study designs across several outcomes precluded meaningful assessment of publication bias and limited the precision, interpretability, and generalisability of the findings, some context‐specific patterns warrant cautious discussion. Overall, the available evidence suggests that participation in FVC interventions is associated with a lower prevalence of food insecurity and higher food consumption scores, within the constraints of the current evidence base. These results align with existing literature (Stewart et al. 2015) and suggest that FVC interventions may support regional progress toward SDG 2 (Zero Hunger) and SDG 12 (Responsible Consumption and Production), particularly when implemented through culturally embedded and system‐wide approaches (Djekic et al. 2021). Our findings are further supported by a recent review highlighting the impact pathways and intervention strategies that link value chain improvements with diet quality (Ridoutt et al. 2019).

Ridoutt et al. (2019) identified 10 impact pathways illustrating the multiple entry points through which FVC interventions can improve food security. These include increasing micronutrient availability through fortification and bio‐fortification and expanding access to nutritious whole foods via diversified production and distribution. Preserving nutritional quality through better processing and packaging and improving access by reducing food losses and transaction costs are also critical. Economic empowerment of value‐chain actors, particularly by enhancing women's access to credit, forms another pathway. Additional pathways include increasing access through public distribution channels such as school meals and targeted food programs; improving the desirability of nutritious foods by ensuring safety and appealing appearance; strengthening nutrition knowledge through education; and enhancing desirability by shaping social perceptions of status and improving convenience, including offering easy‐to‐prepare portioned foods (Ridoutt et al. 2019).

However, in this study, most FVC interventions in sub‐Saharan Africa focused primarily on diversifying agricultural practices through food production, nutrition‐sensitive approaches, economic empowerment (such as improving women's access to credit), and public distribution programmes, including school meals and food aid targeted at vulnerable individuals and households. This relatively narrow focus may limit the external validity of our findings and reflects several contextual and structural factors. Compared to other regions, food processing and distribution technologies in SSA lag significantly due to inadequate investment in infrastructure, technological innovation, and skilled labour for processing and quality control (Bjornlund et al. 2022; Ogutu et al. 2024; Ritchie 2022; Wudil et al. 2022). Additionally, weak and often competing land tenure policies negatively impact food production (Bjornlund et al. 2022; Ogutu et al. 2024; Ritchie 2022; Wudil et al. 2022). Poor investment in essential infrastructure—including unreliable electricity, limited access to water, and insufficient food and transportation networks‐ contributes to substantial post‐harvest losses (Ogutu et al. 2024). Limited access to modern technical advancements further hinders food production, as farmers struggle to adopt innovative processing methods, automated farming equipment, and digital tools that could improve efficiency, sustainability, and food safety (Ogutu et al. 2024; Wudil et al. 2022). Moreover, long‐term investments in agricultural improvements and digital technologies across the FVC are unlikely to gain traction without secure land tenure systems, robust food policy frameworks, and effective regulatory mechanisms (Bjornlund et al. 2022).

Our findings indicate no clear associations between FVC interventions and children's HAZ or WHZ, while a modest positive association was observed for WAZ. Although many FVC frameworks and causal pathways conceptually link FVC interventions with improvements in child nutritional status, empirical evidence to support these relationships remains limited (Nicholson et al. 2021; Nordhagen 2020; Ridoutt et al. 2019). This scarcity of evidence may stem from various factors that negatively affect multiple stages of FVC interventions. For example, cultural food beliefs and taboos, which influence how food is shared within households, are often not addressed by these interventions. Such socio‐cultural practices include the status attributed to animal‐based foods, cultural attitudes toward certain foods, methods of preparation and sharing, and feeding norms. The ‘eat first’ paradigm, traditionally prioritizing the male breadwinner as the primary financial provider, reflects deeply ingrained gendered hierarchies in food distribution that can negatively affect the nutritional status of women and children (Atreya et al. 2024). These gendered hierarchies, frequently based on the ‘eat first’ model, differ among SSA cultures but typically lead to disparities in food access within families. FVC initiatives need to be incorporated into local food systems and include culturally sensitive nutrition approaches that consider these dynamics.

Furthermore, FVC interventions in SSA operate within highly complex socio‐cultural, political, economic, and regulatory environments that can shape their effects on children's nutrition (Assan 2023). As the region confronts the triple burden of malnutrition including undernutrition, overnutrition, and micronutrient deficiencies (Christian and Dake 2022), concerns have emerged that interventions emphasizing food processing to expand the availability of low‐cost products may unintentionally worsen nutritional outcomes. Debates on ultra‐processed foods in the region often overlook traditional food processing, focusing instead on Western industrial products high in sugar, sodium, and saturated fat. This narrow framing risks misclassifying culturally embedded methods such as fermentation, drying, and salting as unhealthy, despite their long‐standing nutritional and safety benefits (Assan 2023). The health effects of processed foods vary with local diets, environments, and processing practices. Without Africa‐specific evidence, it remains difficult to assess how ultra‐processed foods influence health in diverse African contexts (Assan 2023; Moyo and Pisa 2025). Existing evidence indicates that many inexpensive processed foods linked to global value chains are nutritionally poor and contribute to rising childhood obesity (Gereffi and Christian 2007; Hashad et al. 2024). Hashad et al. (2024) showed increasing overweight and obesity in LMICs engaged in backward FVC markets (inputs and services that need to be imported to produce goods for export), whereas participation in forward FVC markets (where exported commodities enter downstream value chains or are re‐exported) had minimal obesity effects, particularly in rural areas.

The available evidence indicates that FVC activities that target all stages of the FVC continuum while leveraging regional food hubs can significantly enhance the skills and capacity of smallholder farmers, strengthen their collaborative networks, and support the operations of small businesses. This integrated approach to FVC development not only improves farmers' income but also helps to reduce food loss and waste across multiple points along the chain, from upstream producer‐led processes to downstream consumer‐driven activities (Nash et al. 2017; Stewart et al. 2015). These results align with our findings, which demonstrate that FVC interventions increase agricultural returns and reduce post‐harvest losses. However, when FVC activities are limited to training alone, their effect on farmers' income appears negligible and statistically non‐significant (Stewart et al. 2015). Furthermore, fragmented FVC interventions, such as those focused solely on post‐harvest practices, show limited success in addressing aflatoxin contamination, which is a major contributor to food waste and health problems (Farkas et al. 2022). This is consistent with our own findings, which show that FVC interventions did not significantly reduce aflatoxin infestation in maize. Collectively, these insights underscore the importance of adopting comprehensive, system‐wide FVC approaches that span the entire value chain, rather than narrowly focused or isolated interventions.

Nonetheless, despite the methodological constraints, the findings may suggest the need for comprehensive policy strategies that support culturally relevant, multi‐phase FVC interventions. While most interventions target food security through measures that incorporate productivity, purchasing power, and economic growth, their broader population health implications remain underexplored (May 2018; Noort et al. 2022). Governments and development partners should invest in infrastructure, land tenure reforms, and nutrition education, while embedding public health measures within FVC initiatives. Priority areas include contextualized nutrition labelling, restrictions on child‐directed marketing, and clearer guidance on processed foods (Abdool Karim et al. 2025; May 2018), all aligned with African dietary practices and local value chains. Without Africa‐specific definitions of processed and ultra‐processed foods, designing culturally appropriate and effective interventions remains challenging. Strengthening policies and regulatory enforcement to address aggressive industry marketing, low consumer nutrition literacy, and weak oversight is essential (Abdool Karim et al. 2025), as neglecting these issues may undermine the health benefits of FVC interventions. Future research should develop Africa‐specific conceptualisations of processed and ultra‐processed foods and prioritize: (1) evidence on how different FVC stages influence nutritional outcomes and population health; (2) assessment of unintended consequences related to processed food availability, affordability, and marketing; and (3) understanding of how socio‐cultural norms, consumer behavior, and regulatory environments shape food choices and health risks. Interdisciplinary research integrating nutrition, economics, systems science, and implementation science is vital for designing FVC interventions that advance food security, economic development, and population health in the region.

## 5 Limitations

This review is subject to several limitations. A key limitation of this review is the small number of studies for certain outcomes (HAZ, WAZ, food production and consumption indicators). This limitation carries two implications. First, Egger's regression tests may be underpowered for outcomes with fewer than 10 studies, increasing the likelihood of false‐negative results. That is, failing to detect publication bias that is present. Second, the small number of studies restricts the statistical power of subgroup analyses, limiting the ability to draw robust conclusions for such outcomes (Ioannidis and Trikalinos 2007). Although the search strategy was designed to be comprehensive, it was limited to English‐language publications, which limits the generalisability of this systematic review. The authors acknowledge that such exclusions may have introduced language bias, particularly as the FVC literature in the regions may also be available in French and Arabic in regions but we did not have a budget for translation. Consequently, our conclusions may not fully capture the diversity of FVC interventions or their impacts across different linguistic and socio‐cultural contexts. Furthermore, most included studies were quasi‐experimental, which may introduce selection bias and limit causal inference. However, data were extracted from propensity score matching models using the nearest‐neighbor matching method, which strengthens causal inference in quasi‐experimental designs by reducing selection bias and constructing more comparable treatment and control groups. Although it does not fully replicate randomization, PSM improves the credibility of estimated effects by balancing observed covariates and isolating the contribution of the intervention (Deng et al. 2023). Nonetheless, while PSM approaches strengthen causal inference, they cannot fully eliminate residual confounding. Unobserved factors, measurement error, and imperfect model specification may still influence estimated associations. Findings from this review should therefore be interpreted as indicative of associations rather than definitive causal effects.

Additionally, heterogeneity in intervention design, sampling frames, and the psychometric properties of assessment tools further contributes to the variability observed across studies (Aleksandrowicz et al. 2017; Mahal et al. 2024; Ruel et al. 2018; Walsh et al. 2020). For food security assessment, commonly used methods such as food‐frequency questionnaires and food balance sheets tend to overestimate food and nutrient intake by approximately 13%–35%, whereas 24‐h dietary recalls tend to underestimate intake by about 9% (Aleksandrowicz et al. 2017). These tools are susceptible to several challenges, including social desirability and recall biases, interviewer burden, and constraints related to time and cost, all of which impact the reliability and validity of data collection and, consequently, the findings and interpretations of studies (Mahal et al. 2024; Walsh et al. 2020). During the data extraction phase, several recurring issues were identified, including typographical errors in reported outcomes, the use of descriptive analyses that fail to control for confounding factors (Omotoso et al. 2024; Walsh et al. 2020), and a heavy reliance on self‐reported measures. These limitations mirror those highlighted in previous literature (Silva et al. 2024). For certain outcomes, such as aflatoxin infestation of maize, the nutrient adequacy ratio, minimum dietary diversity, the quantity of food stored for future use, and legume and tuber production, a meta‐analysis was conducted based on a single study. Such instances inherently lack the robustness of data pooling, making it impossible to assess heterogeneity or derive broadly generalisable conclusions. The pervasive use of self‐reported outcome measures remains a significant concern, as participants' responses may be influenced by their subjective perceptions of the intervention. This can lead to inflated estimates of effectiveness, thereby affecting the credibility of the reported impact of FVC interventions. Finally, the interventions reviewed were highly heterogeneous in design, scope, and implementation context, contributing to substantial statistical heterogeneity across outcomes.

## 6 Policy Implication and Conclusion

Despite the methodological constraints, this systematic review included studies from diverse settings and ecological zones across SSA, many with large sample sizes. Findings should be interpreted in light of the predominantly quasi‐experimental evidence base, with limited randomized evidence available. Overall, FVC interventions appear to be associated with improvements in food security indicators, particularly economic and dietary outcomes, including higher agricultural returns, increased food expenditure, improved food consumption scores, and greater dietary diversity. However, the benefits of FVC interventions are not uniform across settings or outcomes. Substantial methodological and contextual heterogeneity was evident across studies, and evidence for effects on child anthropometric outcomes, food stability, and food safety indicators was limited, inconsistent, or inconclusive. While subgroup analyses suggest differential effects across FVC stages for HDDS, these findings warrant cautious interpretation given the substantial between‐study heterogeneity. Moreover, there is growing concern that such interventions may be linked to increases in overweight and obesity among children and women. This highlights the need for comprehensive, well‐coordinated food policies and regulatory frameworks across the food governance system to mitigate potential risks. To maximize the benefits of FVC interventions, their design and implementation should be grounded in co‐creation, actively engaging producers, distributors, retailers, and consumers. Interventions should be tailored to local food systems, market structures, and socio‐cultural norms, rather than adopting one‐size‐fits‐all approaches that risk overlooking the contextual factors most likely to determine effectiveness. Culturally competent interventions that integrate local practices and food value beliefs can enhance community support, uptake, and scalability. Such approaches are critical for addressing complex sociocultural, economic, and environmental factors, as well as entrenched inequities in food availability, access, and utilization. Therefore, while FVC interventions offer wide‐ranging benefits, there is an urgent need for transformative policy and regulatory reforms across the entire FVC continuum. Evidence from this review indicates that impacts are stage‐specific and highly dependent on implementation context, with more consistent benefits observed for income, market participation, and dietary diversity than for anthropometric or food stability outcomes. Accordingly, scaling FVC interventions across sub‐Saharan Africa should be selective and context‐responsive, prioritizing value chain stages, population groups, and institutional environments where effectiveness has been demonstrated, rather than pursuing indiscriminate regional expansion. These reforms should consider socio‐cultural and economic determinants, environmental shocks, and the fragmented nature of food production and processing infrastructure. Policies promoting flexibility in infrastructure investment, technological innovation, and regulatory oversight are essential, as is harmonization of food processing and trading regulations within and between countries. Integrated food markets and trade linkages, alongside robust food safety standards, are necessary to realize the full potential of FVC interventions in SSA.

## Author Contributions

**Ramadhani Noor:** writing – review and editing, validation, resources, funding acquisition. **Rashid Abdulai:** funding acquisition, validation, resources, writing – review and editing. **Stanley Chitekwe:** conceptualization, funding acquisition, methodology, writing – review and editing, software, supervision, resources. **Sanjay Kumar Das:** writing – review and editing, validation, funding acquisition. **Kh Shafiur Rahaman:** investigation, methodology, validation, software, data curation, writing – review and editing, project administration. **Andre M. N. Renzaho:** conceptualization, investigation, methodology, validation, visualization, software, formal analysis, project administration, supervision, data curation, writing – original draft, writing – review and editing.

## Funding

The authors have nothing to report.

## Ethics Statement

The authors have nothing to report.

## Consent

All authors have read and agreed to the published version of the manuscript.

## Conflicts of Interest

The authors declare no conflicts of interest.

## Supporting information


**Figure S1:** FVC interventions' impact on anthropometric measures.
**Figure S2:** FVC interventions' impact on food composition scores.
**Figure S3:** FVC interventions' impact on dietary diversity score.
**Figure S4:** FVC interventions' impact on agricultural return per hectare in USD.

**Table S1:** fsn371881‐sup‐0002‐TableS1.docx.

**Table S2:** fsn371881‐sup‐0003‐TableS2.docx.

## Data Availability

All data are available within the paper.
