# Supplementary material for: The Effect of Food Value Chain Interventions on Food Security in Sub‐Saharan Africa: A Systematic Review and Meta‐Analysis
Source: Food Sci Nutr. 2026 Jun 15;14(6):e71881. doi: 10.1002/fsn3.71881 (PMC13269678; doi:10.1002/fsn3.71881)
Supplement: Supplementary file 1 — Figure S1: FVC interventions' impact on anthropometric measures. Figure S2: FVC interventions' impact on food composition scores. Figure S3: FVC interventions' impact on dietary diversity score. Figure S4: FVC interventions' impact on agricultural return per hectare in USD. [file FSN3-14-e71881-s003.pdf]

Supplementary figure 1: FVC interventions' impact on anthropometric measures

1.1. Height-for-age

1.1.1. HAZ by study design

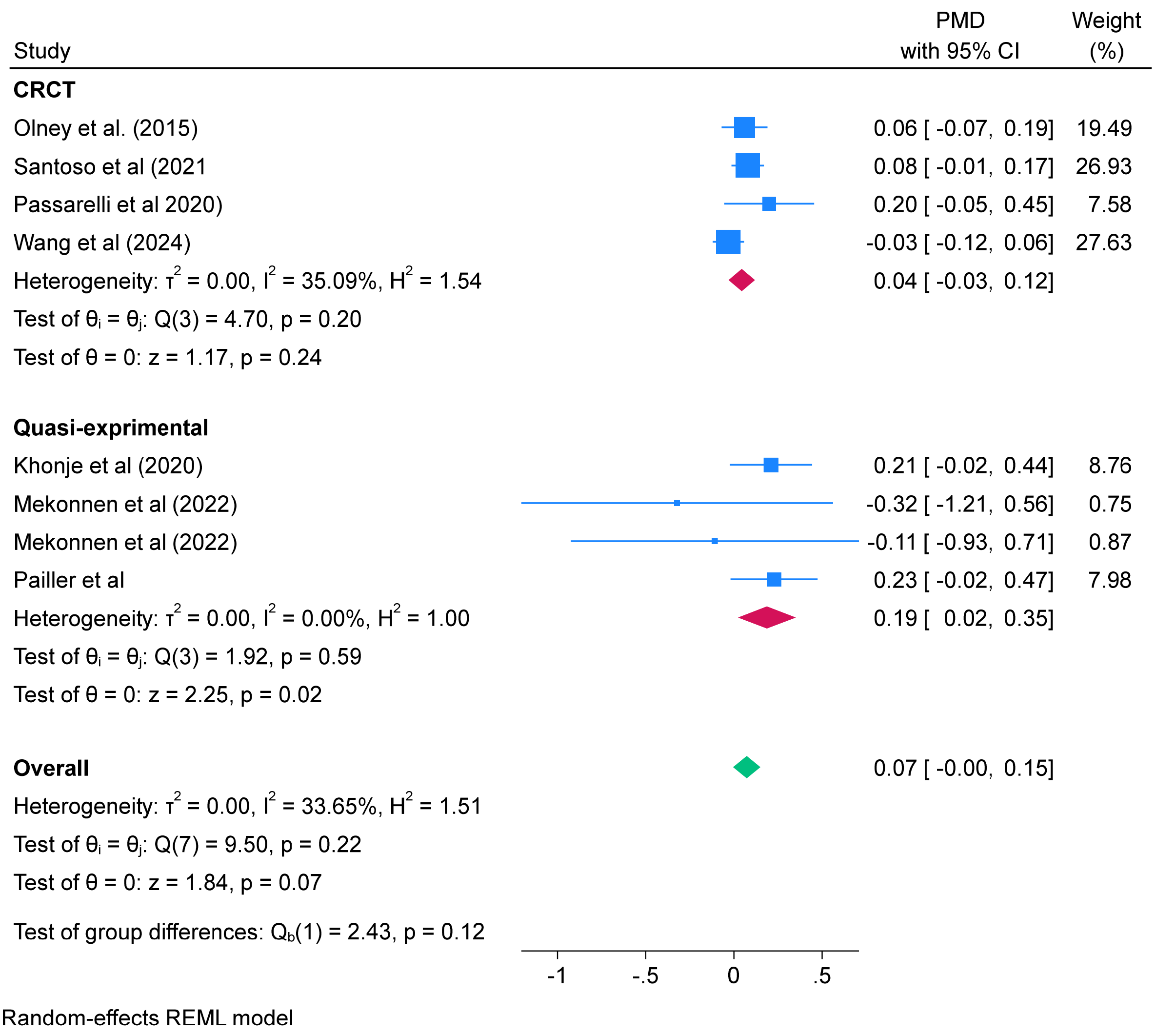

1.1.2. HAZ by region

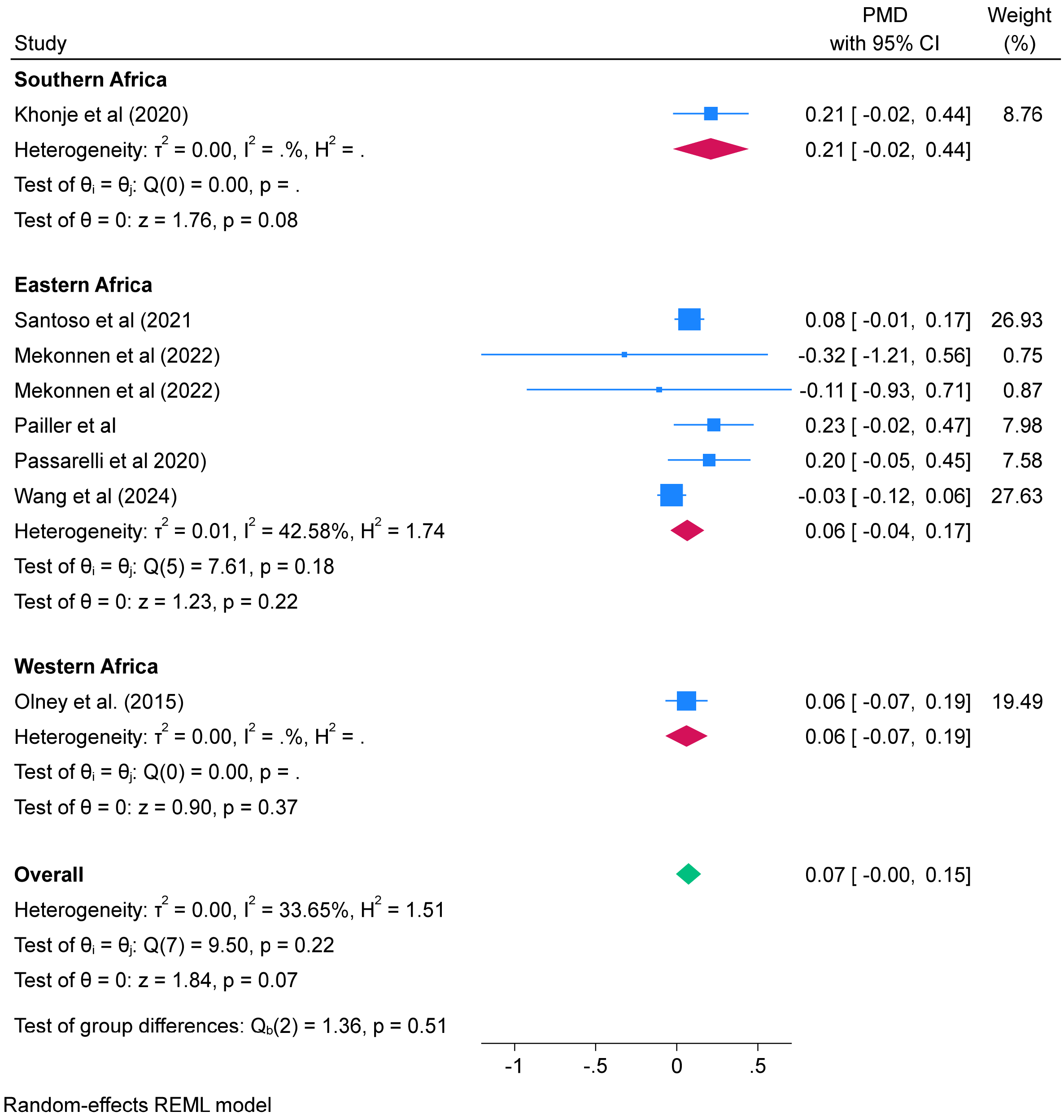

1.1.3. HAZ by FVC stage

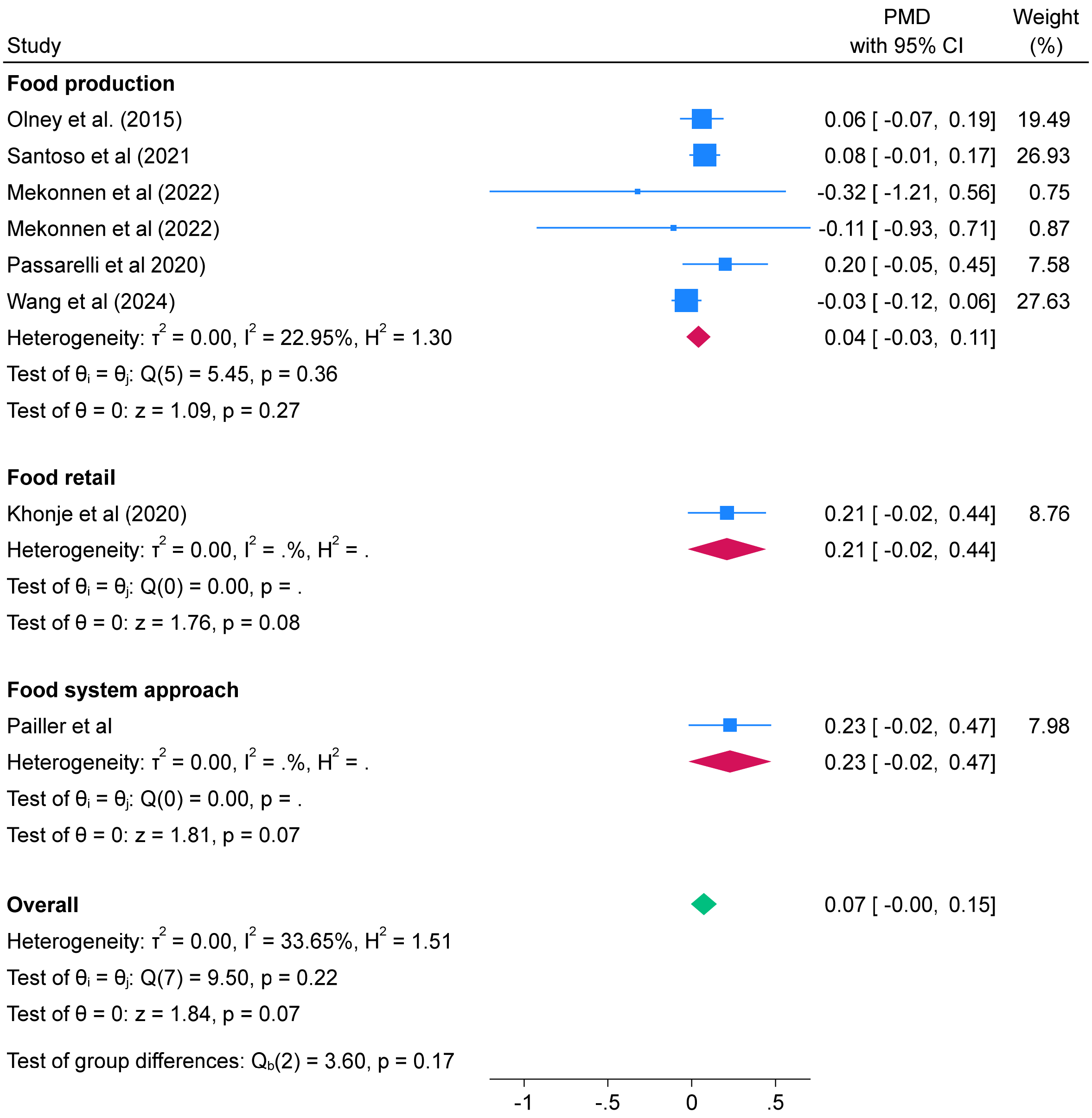

1.1.4. Random-effects REML model

1.2. Weight-for-age

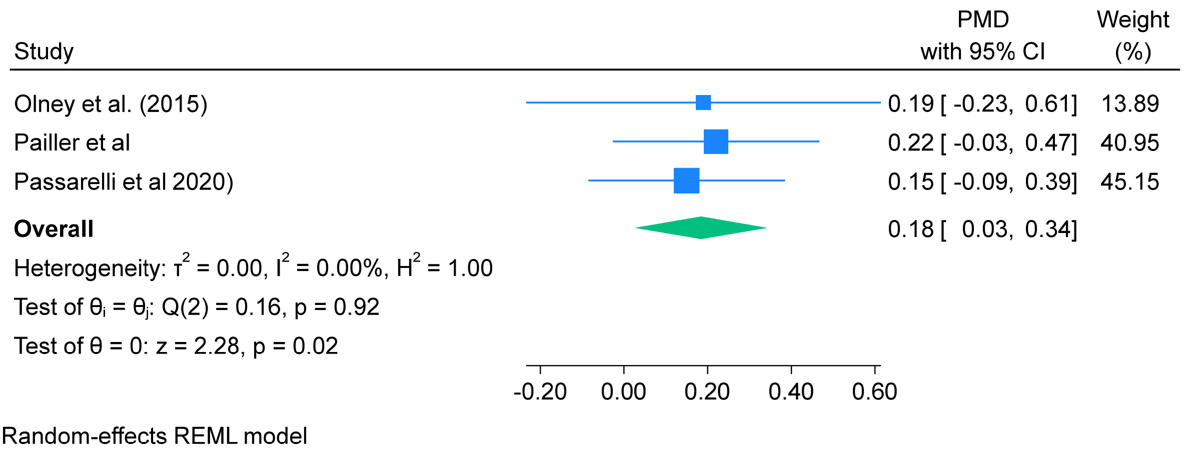

1.3. Weight-for-height

1.3.1. WHZ by study design

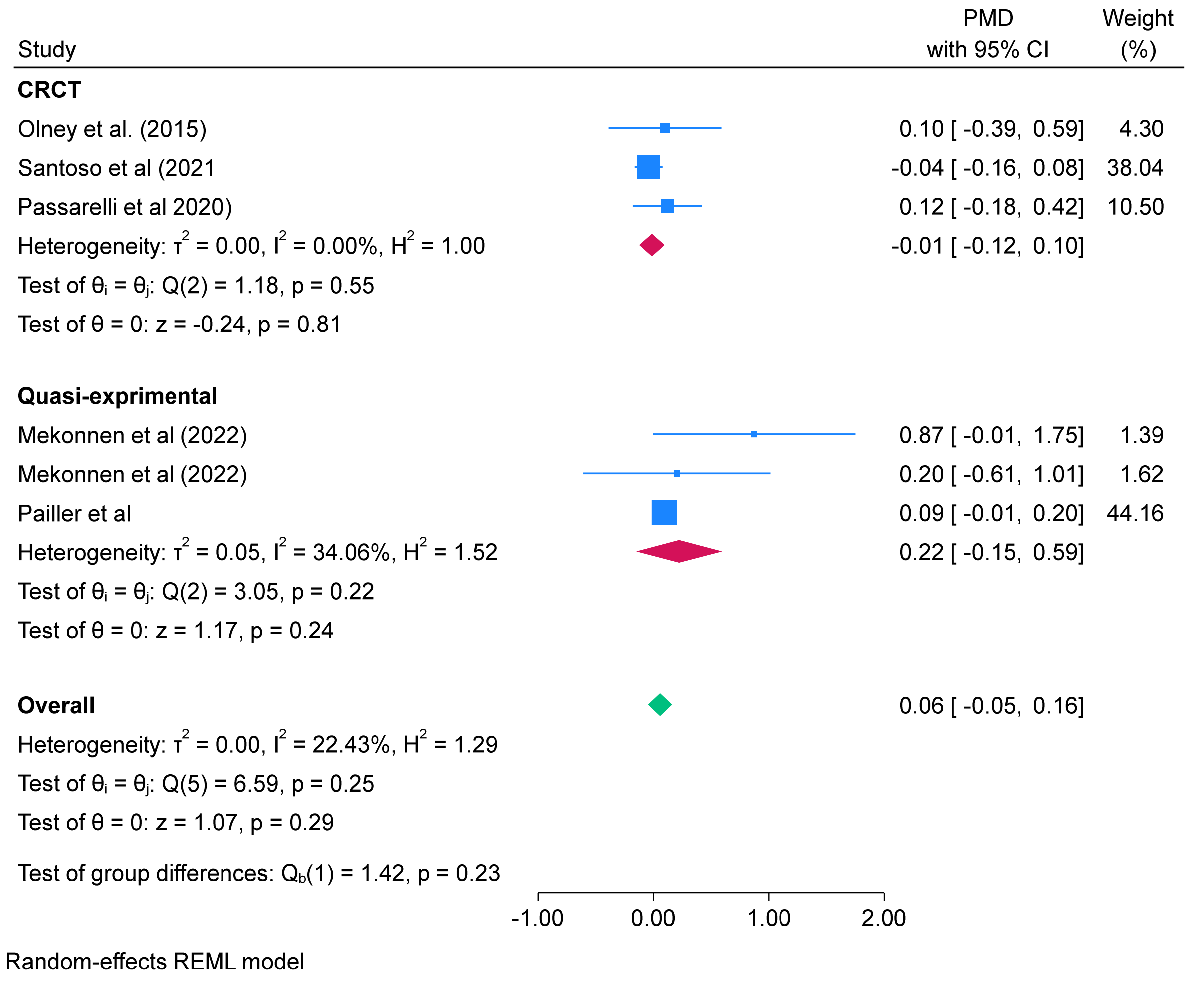

1.3.2. WHZ by region

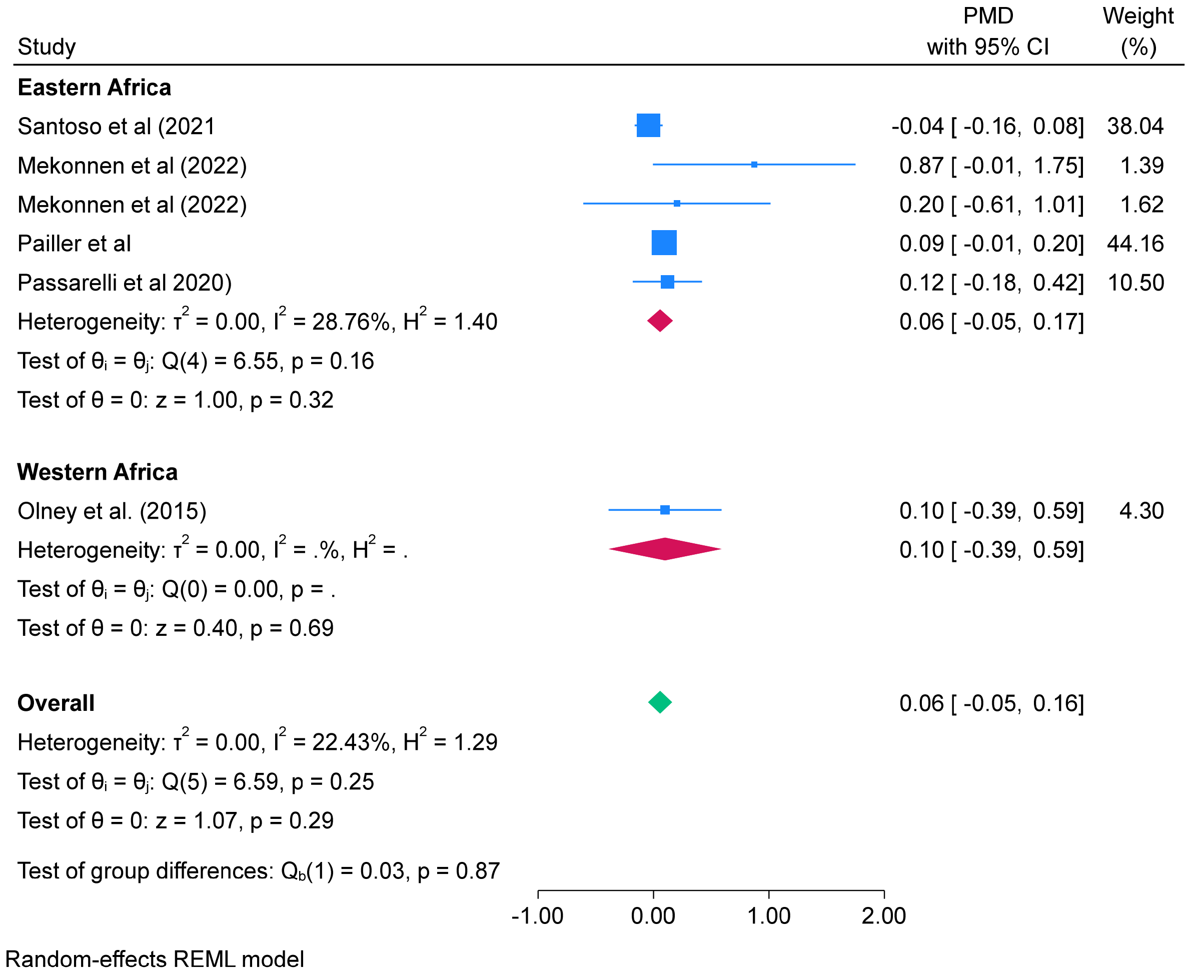

1.3.3. WHZ by FVC stage

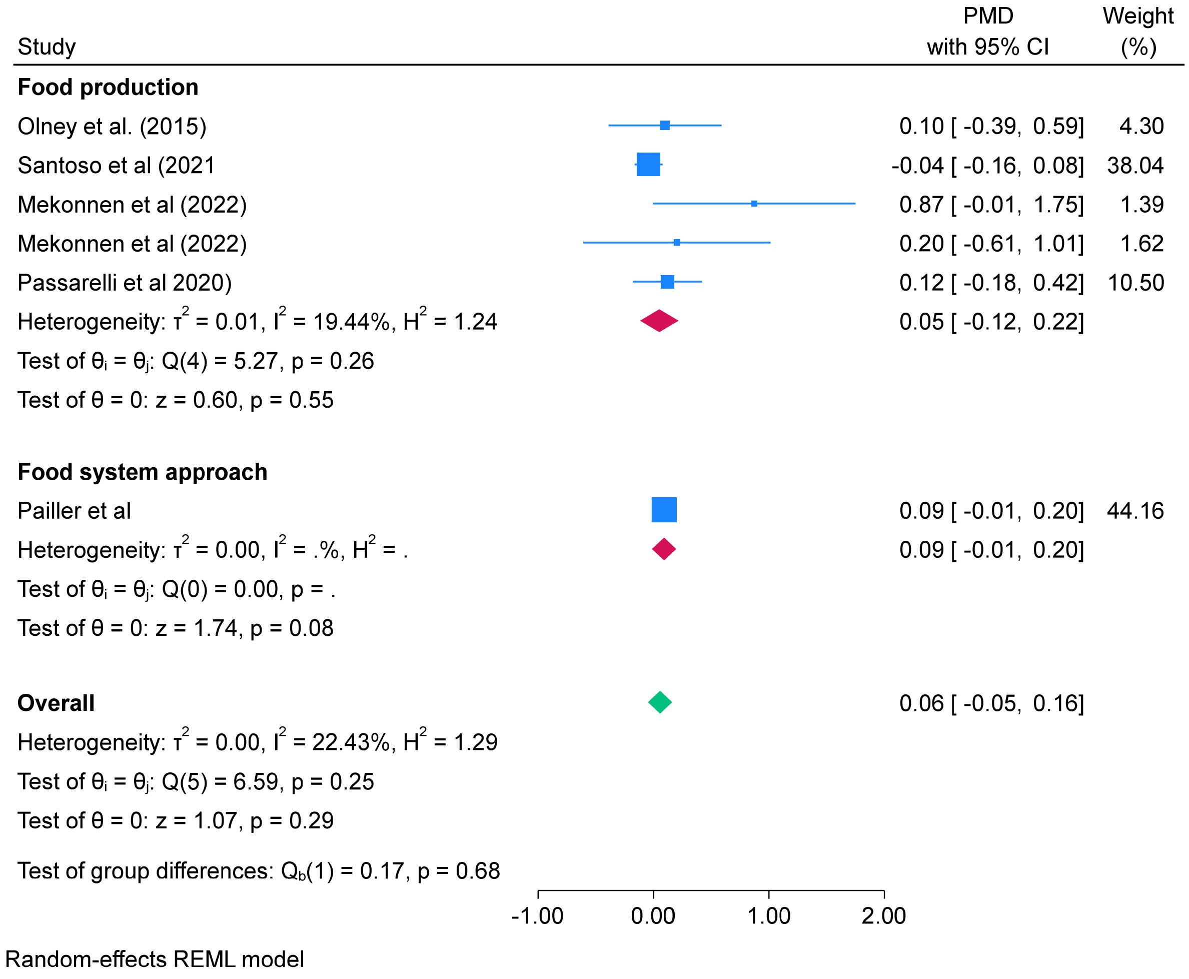

Supplementary figure 2: FVC interventions' impact on food composition scores

2.1. FCS by region

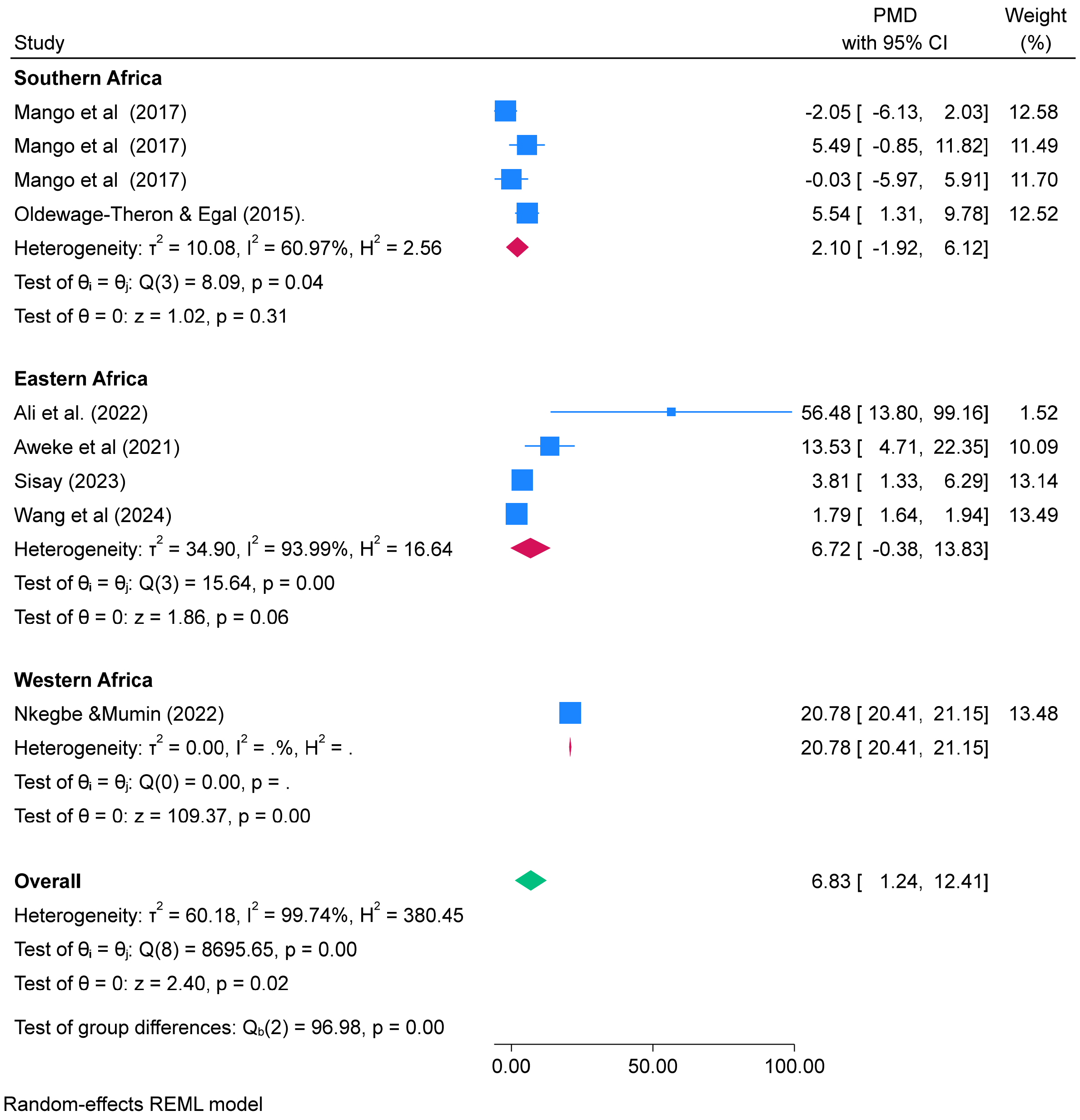

2.2. FCS by study design

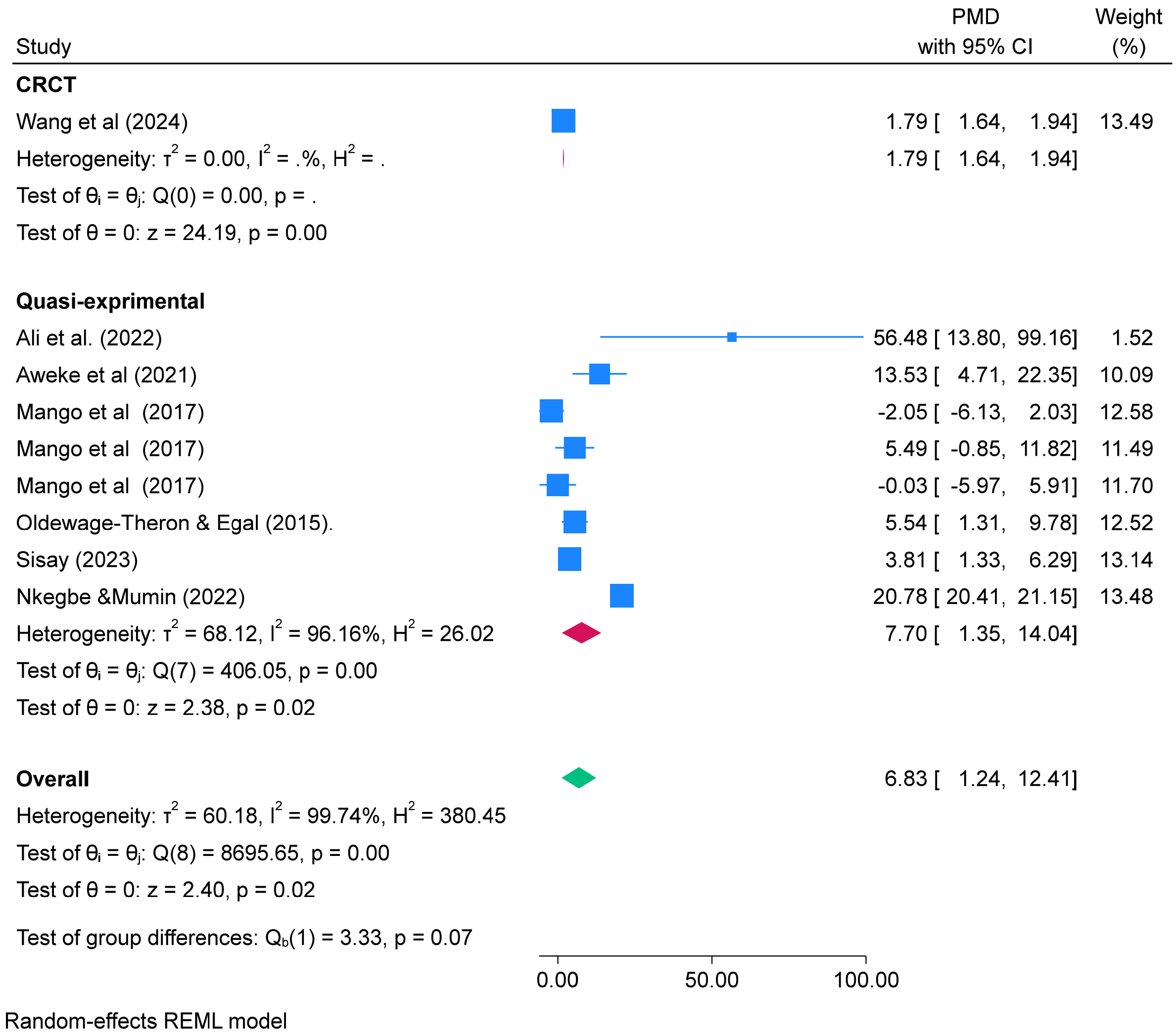

2.3. FCS by FVC stage

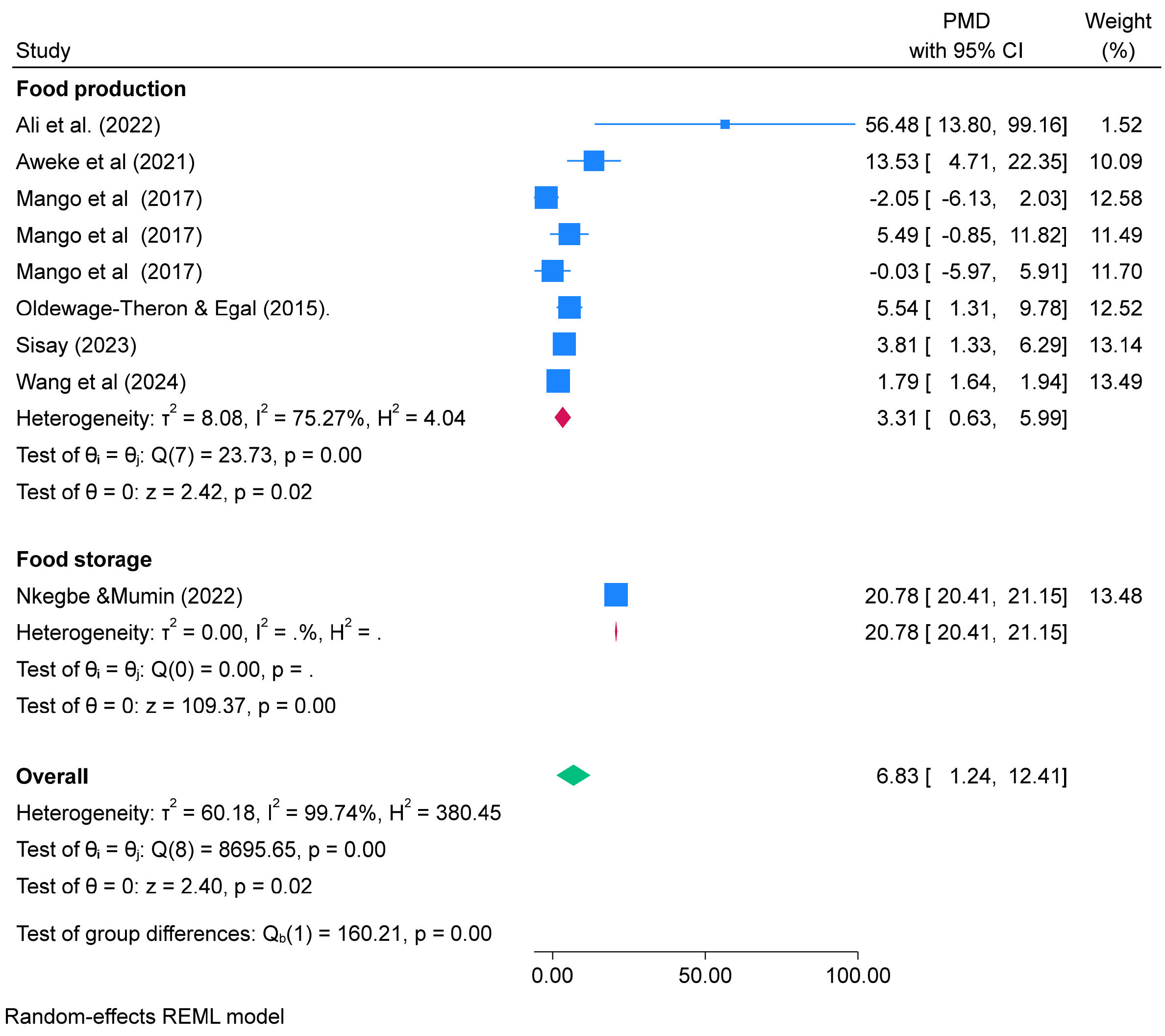

Study
PMD with 95% CI
Weight (%)
Food production
Ali et al. (2022) 56.48 [13.80, 99.16] 1.52
Aweke et al (2021) 13.53 [4.71, 22.35] 10.09
Mango et al (2017) -2.05 [-6.13, 2.03] 12.58
Mango et al (2017) 5.49 [-0.85, 11.82] 11.49
Mango et al (2017) -0.03 [-5.97, 5.91] 11.70
Oldewage-Theron & Egal (2015). 5.54 [1.31, 9.78] 12.52
Sisay (2023) 3.81 [1.33, 6.29] 13.14
Wang et al (2024) 1.79 [1.64, 1.94] 13.49
Heterogeneity: τ^2 = 8.08, I^2 = 75.27%, H^2 = 4.04 3.31 [0.63, 5.99]
Test of $\theta_i = \theta_j$: Q(7) = 23.73, p = 0.00
Test of θ = 0: z = 2.42, p = 0.02
Food storage
Nkegbe &Mumin (2022) 20.78 [20.41, 21.15] 13.48
Heterogeneity: τ^2 = 0.00, I^2 = .%, H^2 = . 20.78 [20.41, 21.15]
Test of $\theta_i = \theta_j$: Q(0) = 0.00, p = .
Test of θ = 0: z = 109.37, p = 0.00
Overall 6.83 [1.24, 12.41]
Heterogeneity: τ^2 = 60.18, I^2 = 99.74%, H^2 = 380.45
Test of $\theta_i = \theta_j$: Q(8) = 8695.65, p = 0.00
Test of θ = 0: z = 2.40, p = 0.02
Test of group differences: $Q_b(1)$ = 160.21, p = 0.00
0.00
50.00
100.00
Random-effects REML model

Supplementary figure 3: FVC interventions' impact on dietary diversity score

3.1. HDDS by region

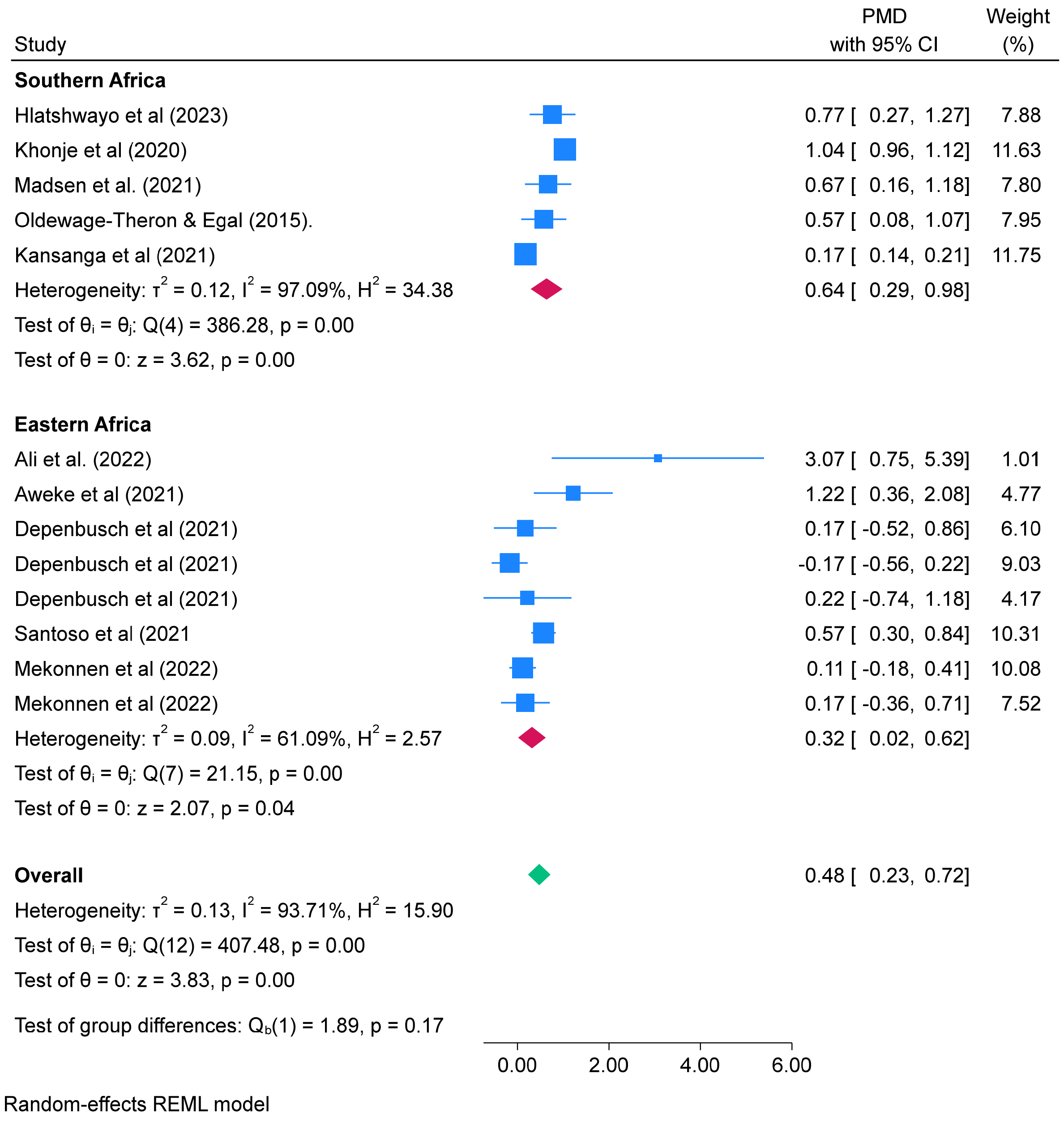

3.2. HDDS by study design

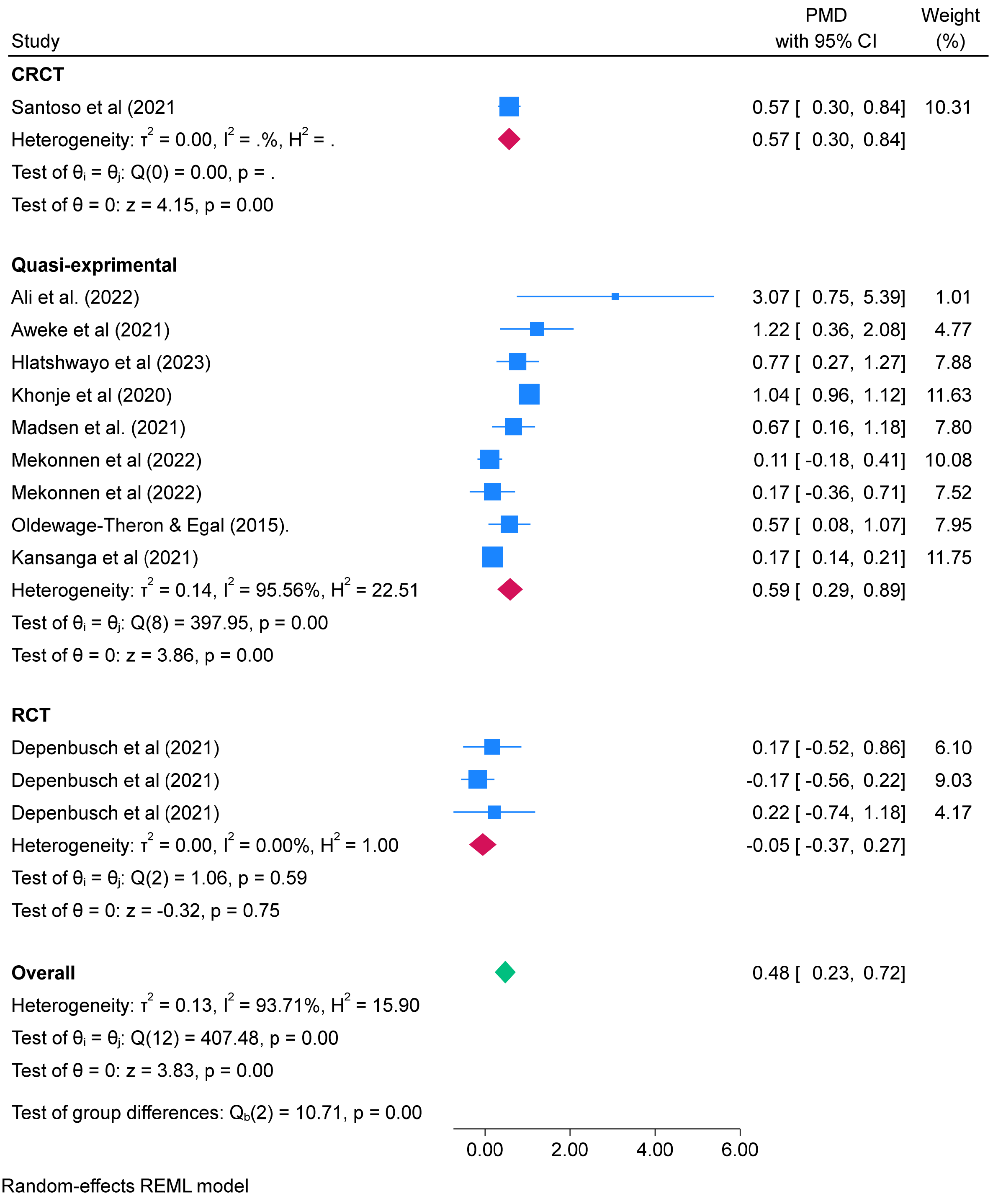

3.3. HDDS by FVC stage

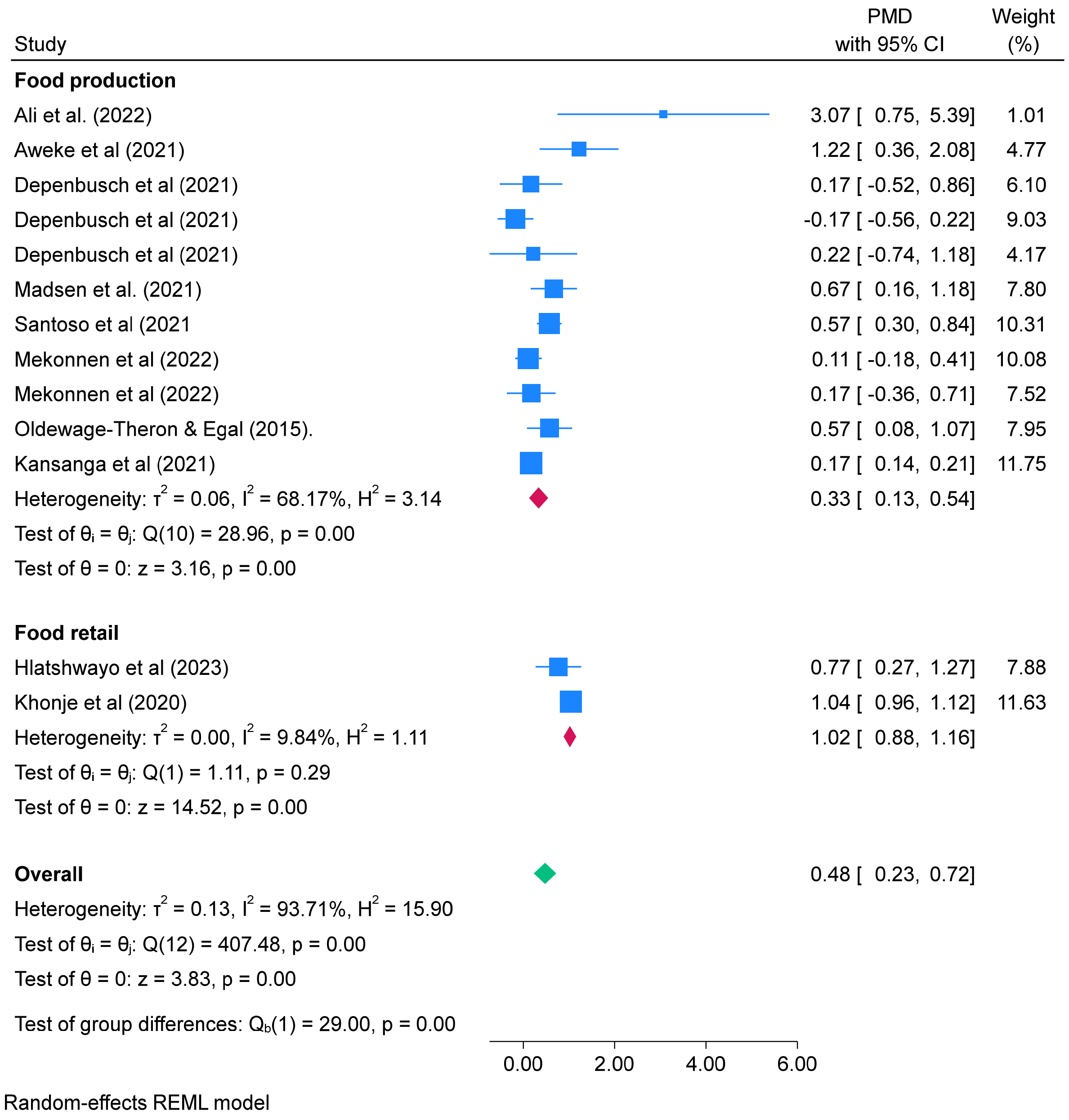

3.4. IDDS

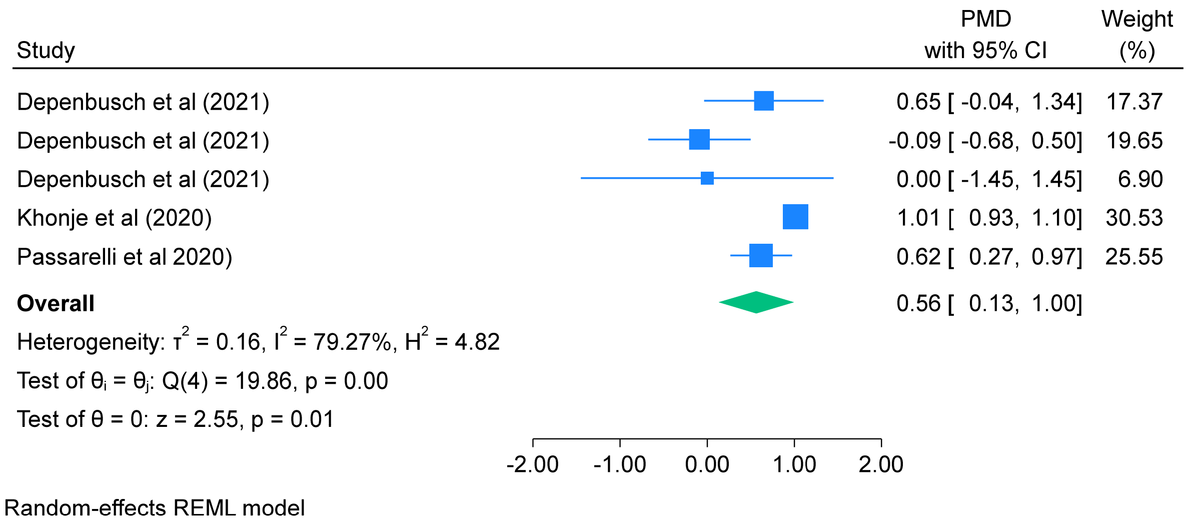
Study
PMD with 95% CI
Weight (%)
Depenbusch et al (2021) 0.65 [-0.04, 1.34] 17.37
Depenbusch et al (2021) -0.09 [-0.68, 0.50] 19.65
Depenbusch et al (2021) 0.00 [-1.45, 1.45] 6.90
Khonje et al (2020) 1.01 [0.93, 1.10] 30.53
Passarelli et al 2020) 0.62 [0.27, 0.97] 25.55
Overall 0.56 [0.13, 1.00]
Heterogeneity: τ^2 = 0.16, I^2 = 79.27%, H^2 = 4.82
Test of $\theta_i = \theta_j$: Q(4) = 19.86, p = 0.00
Test of θ = 0: z = 2.55, p = 0.01
-2.00 -1.00 0.00 1.00 2.00
Random-effects REML model

Supplementary figure 4: FVC interventions' impact on agricultural return per hectare in USD

4.1: by African region

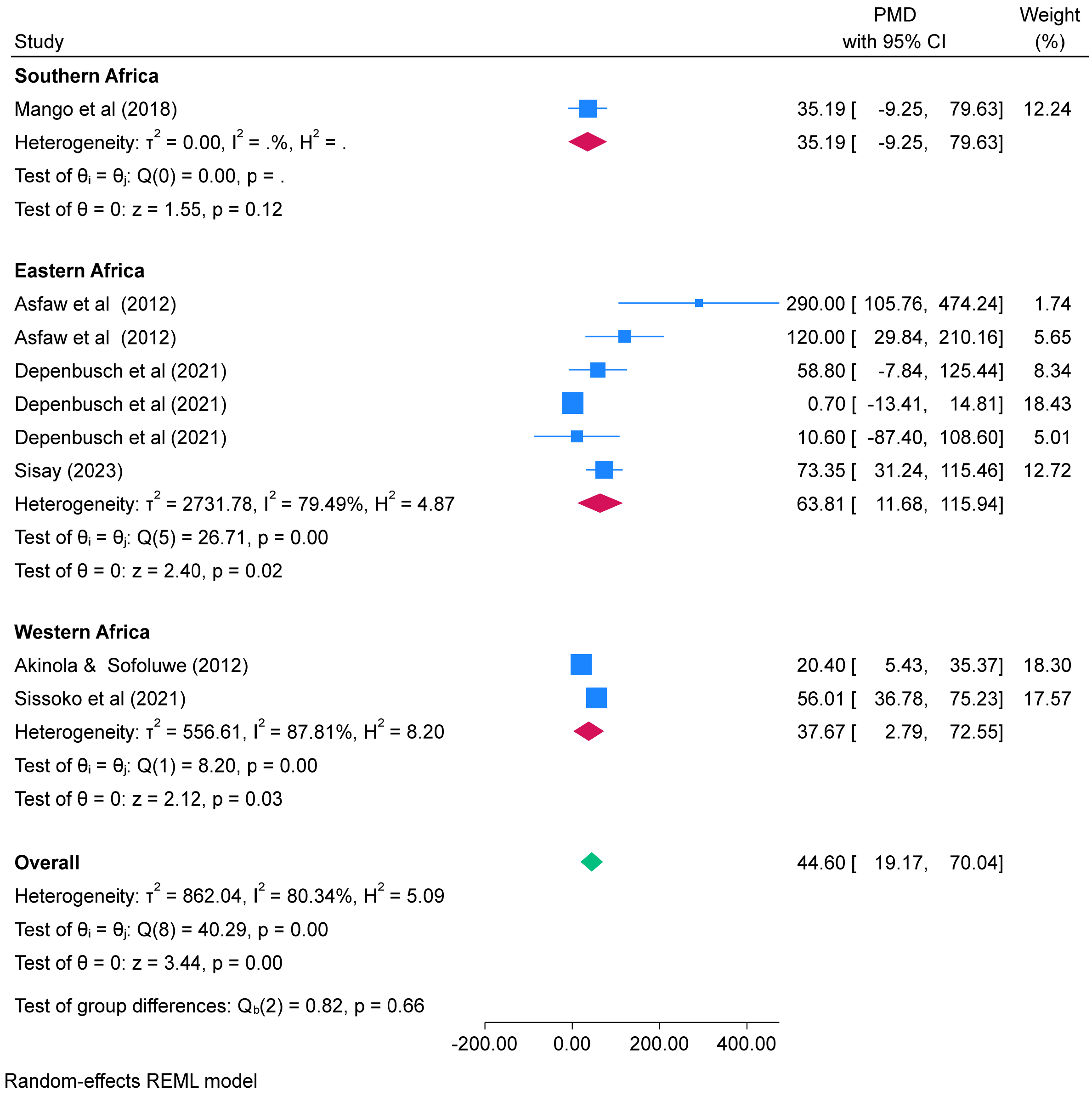

5.2. by study design

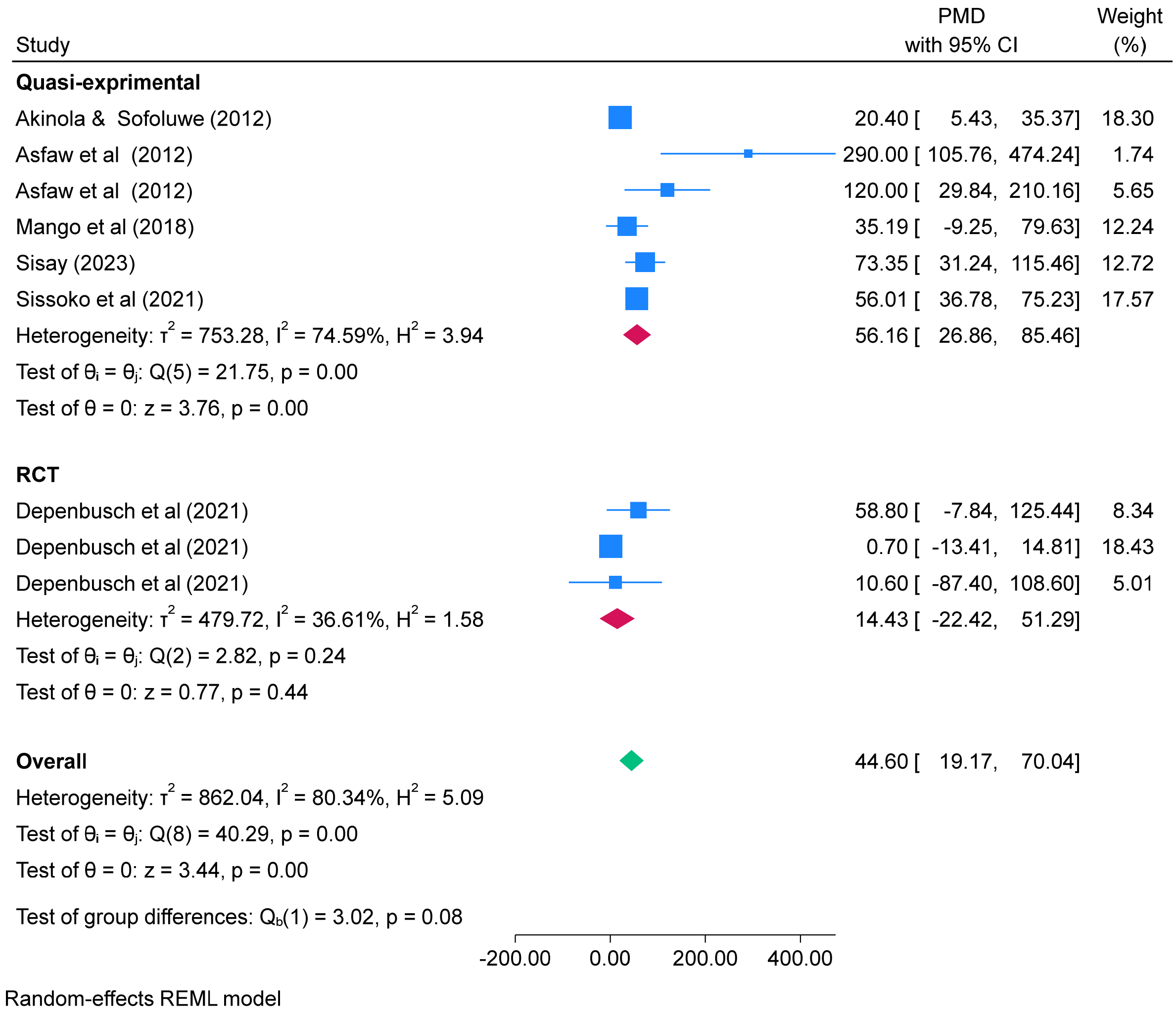

5.3. by FVC stage

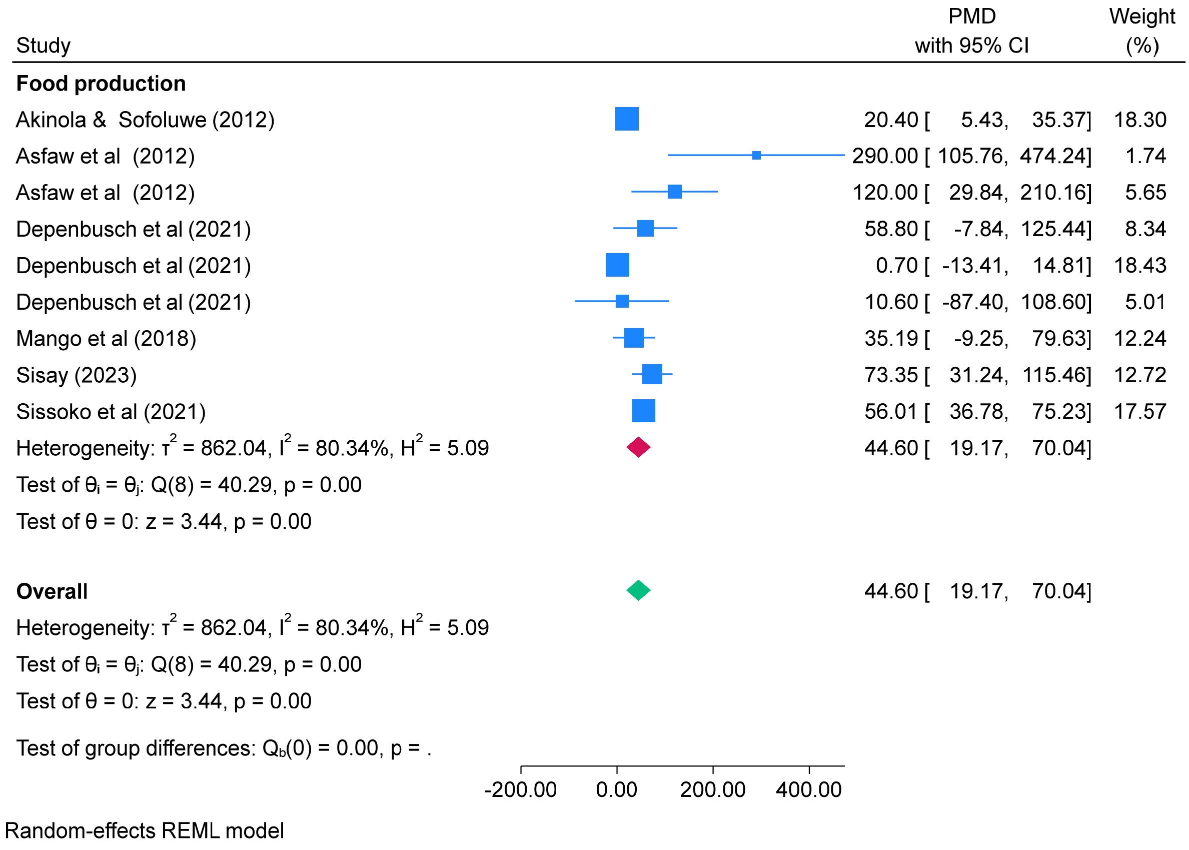

Study
PMD with 95% CI
Weight (%)
Food production
Akinola & Sofoluwe (2012) 20.40 [5.43, 35.37] 18.30
Asfaw et al (2012) 290.00 [105.76, 474.24] 1.74
Asfaw et al (2012) 120.00 [29.84, 210.16] 5.65
Depenbusch et al (2021) 58.80 [-7.84, 125.44] 8.34
Depenbusch et al (2021) 0.70 [-13.41, 14.81] 18.43
Depenbusch et al (2021) 10.60 [-87.40, 108.60] 5.01
Mango et al (2018) 35.19 [-9.25, 79.63] 12.24
Sisay (2023) 73.35 [31.24, 115.46] 12.72
Sissoko et al (2021) 56.01 [36.78, 75.23] 17.57
Heterogeneity: τ^2 = 862.04, I^2 = 80.34%, H^2 = 5.09 44.60 [19.17, 70.04]
Test of $\theta_i = \theta_j$: Q(8) = 40.29, p = 0.00
Test of θ = 0: z = 3.44, p = 0.00
Overall 44.60 [19.17, 70.04]
Heterogeneity: τ^2 = 862.04, I^2 = 80.34%, H^2 = 5.09
Test of $\theta_i = \theta_j$: Q(8) = 40.29, p = 0.00
Test of θ = 0: z = 3.44, p = 0.00
Test of group differences: $Q_b(0)$ = 0.00, p = .
-200.00 0.00 200.00 400.00
Random-effects REML model
