# Supplementary material for: The Effect of Food Value Chain Interventions on Food Security in Sub‐Saharan Africa: A Systematic Review and Meta‐Analysis
Source: Food Sci Nutr. 2026 Jun 15;14(6):e71881. doi: 10.1002/fsn3.71881 (PMC13269678; doi:10.1002/fsn3.71881)
Supplement: Supplementary file 2 — Table S1: fsn371881‐sup‐0002‐TableS1.docx. [file FSN3-14-e71881-s002.docx]

**Ovid MEDLINE(R) ALL**

1 (program* or polic* or strateg* or legislation* or law* or intervention* or technique* or planning or practice* or fiscal or regulation* or sustainable or tax* or subsid* or procurement* or incentive* or Impact* or effect* or evaluat* or impact evaluat* impact assessment or assess or assessing or assessment or analyze or analyse or analyzing or analysing or analysis or analytical or estimate or estimating or estimation or cause or causal or impact* or evaluat* or effect* or experiment* or trial or random* or quasi* or natural experiment* or discontinuity or fixed effect* or regression or difference in differences or instrumental variable* or matching or inverse propensity weight).ti. 7757972

2 ("land tenure" or "property right*" or "land right*" or "property right*" or "land property right" or "land tenure security" or regulation or "land valuation" or "land taxation" or "land size" or "land access" or "documented legal right*" or "de facto land possession" or "land registration" or "land certification" or "land tenure regulari*" or "land reform" or "customary land tenure" or "land governance" or conversion or freehold* or titl* or codification or recognition or customary or certification or right* or "farmers' right*" or "land expansion" or "land registration" or "land information").ti. 539198

3 ("value chain" or value-chain or "value crop*" or "value-crop*" or "market access" or harvesting or storage or "waste disposal" or "food storage" or "grain storage silo" or "value chain development" or "food processing cost" or "agriculture cooperatives" or "marketing cooperatives" or "farmer cooperative" or "agricultural marketing cooperative" or "agriculture coop" or "agri-coop" or "marketing coop" or "agriculture co-op" or "cooperative societies" or subsid* or "input support*" or "credit support*" or "agricultural technical support" or "improved market access" or "food processing" or "diversified income sources" or "distance to market" or extension or "organisation membership*" or "agricultural commercialization" or "livelihood system" or "market access" or "farm to market transport infrastructure" or "output market information" or "transport network*" or "food quality" or "food spoilage" or "marketing cost*" or "transport cost*" or "market information" or "food price*" or "market trade*" or "market demand*" or "export transport" or "domestic transport" or transport* or road* or bridge* or waterway* or boat* or rail* or "train tracks" or ports or airport* or "border crossing*" or "distribution facilit*" or highway* or distribut* or freight or access* or connect* or rural or electric* or power or transmission or grid or energy or "information dissemination" or "communication technolog*" or "information technolog*" or "information service*" or "social network*" or "peer network*" or "commodity exchange" or market* or trader* or "processor*" or "agro-exporter*" or "agroexporter*" or "dealer*" or "agro-dealer*" or "agrodealer*" or "wholesaler*" or "miller*" or "village merchant*" or "commission agent*" or "broker*" or "retailer*" or "aggregator*" or "buyer*" or "trading firm*" or "farm-firm*" or "tender*" or "service provider*" or "trucker*" or "transporter*" or "logistics enterprise*" or "third-party logistics" or "freight forwarder*" or "food manufacturer*" or "packager*" or "distributor* commit*" or contract* or "agreement* fixed pric*" or "arrang*" or "guarant*" or "innovative marketing channel*" or "new marketing channel*" or commercialization or commercialisation or "high-value chain*" or "high-value market*" or "high value agricultur*" or "agrifood transformation*" or "agri-food transformation*" or "e-commerce" or "market reform*" or "market linkage*" or "online agricultural platform*" or "agri-platform*" or agriplatform* or "market hub*" or "internet kiosk*" or storage or waste or "waste management" or preservation or "storage device" or "preservation technique" or "post-harvest loss" or "income generation" or "bio energy" or bio-energy or "contract farming" or "food production" or "Crop cultivation" or livestock raising or aquaculture or "Harvesting" or "Timing of harvest" or "harvesting technique*" or drying or cleaning or Heating or Canning or Drying or dehydration or Juicing or Fermentation or Freezing or "Modified atmosphere packaging" or Pasteurisation or Smoking or Additives or "Pulsed electric fields technology" or "pickling of sugaring" or "Post-Harvest Handling" or Sorting or grading or packaging or storage or vacuuming or milling or Distribution or Transportation or logistics or "supply chain" or management or "Marketing and Retail" or "Wholesale markets" or supermarkets or "farmers' markets" or "online platforms" or "Consumption" or "Food preparation" or cooking or "consumption by end-users").ti. 2190800

4 ("Agricultural marketing" or "marketing regulation" or "agricultural marketing law*" or "agriculture regulation" or "agriculture legislation" or "food standards" or "food regulation" or "food safety" or "food safety standard*" or "food grading" or "food price" or "food price regulation" or "term of trade" or "trading practice*" or condition or trade or "unfair trade" or "unfair trade practice*" or "price control*" or "export support*" or "tax concession*" or quota* or tariff* or "import control*" or "crop regulation" or "price support*" or "crop control*" or "market regulation").ti. 45070

5 2 or 3 or 4 2741926

6 ("nutrition* outcome*" or "nutrition* status" or "diet* diversi*" or micronutrient* or anthropom* or food* or macronutrient* or nutrition* or "food consumption*" or diet* or "food secur*" or "food insecur*" or "food security governance" or "governance for food security").ti. 562921

7 (((Sub-saharan Africa or Central Africa or Eastern Africa or Southern Africa or Western Africa or Angola or Benin or Botswana or Burkina Faso or Burundi or Cabo Verde or Cameroon or Central African Republic or Chad or Comoros or Congo or Democratic Republic of Congo or Republic of congo or Cote d'Ivoire or Equatorial Guinea or Eritrea or Eswatini or Swaziland or Ethiopia or Gabon or Gambia or Ghana or Guinea or Guinea-Bissau or Kenya or Lesotho or Liberia or Madagascar or Malawi or Mali or Mauritania or Mauritius or Mozambique or Namibia or Niger or Nigeria or Rwanda or Sao Tome) and Principe) or Senegal or Seychelles or Sierra Leone or Somalia or South Africa or South Sudan or Sudan or Tanzania or Togo or Uganda or Zambia or Zimbabwe).ti. 72727

8 1 and 5 and 6 and 7 34

9 limit 8 to (english language and yr="2000 - 2024") 32

**Embase Classic+Embase <1947 to 2025 November 14>**

1 (program* or polic* or strateg* or legislation* or law* or intervention* or technique* or planning or practice* or fiscal or regulation* or sustainable or tax* or subsid* or procurement* or incentive* or Impact* or effect* or evaluat* or impact evaluat* impact assessment or assess or assessing or assessment or analyze or analyse or analyzing or analysing or analysis or analytical or estimate or estimating or estimation or cause or causal or impact* or evaluat* or effect* or experiment* or trial or random* or quasi* or natural experiment* or discontinuity or fixed effect* or regression or difference in differences or instrumental variable* or matching or inverse propensity weight).ti. 10282861

2 ("land tenure" or "property right*" or "land right*" or "property right*" or "land property right" or "land tenure security" or regulation or "land valuation" or "land taxation" or "land size" or "land access" or "documented legal right*" or "de facto land possession" or "land registration" or "land certification" or "land tenure regulari*" or "land reform" or "customary land tenure" or "land governance" or conversion or freehold* or titl* or codification or recognition or customary or certification or right* or "farmers' right*" or "land expansion" or "land registration" or "land information").ti. 652503

3 ("value chain" or value-chain or "value crop*" or "value-crop*" or "market access" or harvesting or storage or "waste disposal" or "food storage" or "grain storage silo" or "value chain development" or "food processing cost" or "agriculture cooperatives" or "marketing cooperatives" or "farmer cooperative" or "agricultural marketing cooperative" or "agriculture coop" or "agri-coop" or "marketing coop" or "agriculture co-op" or "cooperative societies" or subsid* or "input support*" or "credit support*" or "agricultural technical support" or "improved market access" or "food processing" or "diversified income sources" or "distance to market" or extension or "organisation membership*" or "agricultural commercialization" or "livelihood system" or "market access" or "farm to market transport infrastructure" or "output market information" or "transport network*" or "food quality" or "food spoilage" or "marketing cost*" or "transport cost*" or "market information" or "food price*" or "market trade*" or "market demand*" or "export transport" or "domestic transport" or transport* or road* or bridge* or waterway* or boat* or rail* or "train tracks" or ports or airport* or "border crossing*" or "distribution facilit*" or highway* or distribut* or freight or access* or connect* or rural or electric* or power or transmission or grid or energy or "information dissemination" or "communication technolog*" or "information technolog*" or "information service*" or "social network*" or "peer network*" or "commodity exchange" or market* or trader* or "processor*" or "agro-exporter*" or "agroexporter*" or "dealer*" or "agro-dealer*" or "agrodealer*" or "wholesaler*" or "miller*" or "village merchant*" or "commission agent*" or "broker*" or "retailer*" or "aggregator*" or "buyer*" or "trading firm*" or "farm-firm*" or "tender*" or "service provider*" or "trucker*" or "transporter*" or "logistics enterprise*" or "third-party logistics" or "freight forwarder*" or "food manufacturer*" or "packager*" or "distributor* commit*" or contract* or "agreement* fixed pric*" or "arrang*" or "guarant*" or "innovative marketing channel*" or "new marketing channel*" or commercialization or commercialisation or "high-value chain*" or "high-value market*" or "high value agricultur*" or "agrifood transformation*" or "agri-food transformation*" or "e-commerce" or "market reform*" or "market linkage*" or "online agricultural platform*" or "agri-platform*" or agriplatform* or "market hub*" or "internet kiosk*" or storage or waste or "waste management" or preservation or "storage device" or "preservation technique" or "post-harvest loss" or "income generation" or "bio energy" or bio-energy or "contract farming" or "food production" or "Crop cultivation" or livestock raising or aquaculture or "Harvesting" or "Timing of harvest" or "harvesting technique*" or drying or cleaning or Heating or Canning or Drying or dehydration or Juicing or Fermentation or Freezing or "Modified atmosphere packaging" or Pasteurisation or Smoking or Additives or "Pulsed electric fields technology" or "pickling of sugaring" or "Post-Harvest Handling" or Sorting or grading or packaging or storage or vacuuming or milling or Distribution or Transportation or logistics or "supply chain" or management or "Marketing and Retail" or "Wholesale markets" or supermarkets or "farmers' markets" or "online platforms" or "Consumption" or "Food preparation" or cooking or "consumption by end-users").ti. 2683978

4 ("Agricultural marketing" or "marketing regulation" or "agricultural marketing law*" or "agriculture regulation" or "agriculture legislation" or "food standards" or "food regulation" or "food safety" or "food safety standard*" or "food grading" or "food price" or "food price regulation" or "term of trade" or "trading practice*" or condition or trade or "unfair trade" or "unfair trade practice*" or "price control*" or "export support*" or "tax concession*" or quota* or tariff* or "import control*" or "crop regulation" or "price support*" or "crop control*" or "market regulation").ti. 55152

5 2 or 3 or 4 3353116

6 ("nutrition* outcome*" or "nutrition* status" or "diet* diversi*" or micronutrient* or anthropom* or food* or macronutrient* or nutrition* or "food consumption*" or diet* or "food secur*" or "food insecur*" or "food security governance" or "governance for food security").ti. 713164

7 (((Sub-saharan Africa or Central Africa or Eastern Africa or Southern Africa or Western Africa or Angola or Benin or Botswana or Burkina Faso or Burundi or Cabo Verde or Cameroon or Central African Republic or Chad or Comoros or Congo or Democratic Republic of Congo or Republic of congo or Cote d'Ivoire or Equatorial Guinea or Eritrea or Eswatini or Swaziland or Ethiopia or Gabon or Gambia or Ghana or Guinea or Guinea-Bissau or Kenya or Lesotho or Liberia or Madagascar or Malawi or Mali or Mauritania or Mauritius or Mozambique or Namibia or Niger or Nigeria or Rwanda or Sao Tome) and Principe) or Senegal or Seychelles or Sierra Leone or Somalia or South Africa or South Sudan or Sudan or Tanzania or Togo or Uganda or Zambia or Zimbabwe).ti. 85482

8 1 and 5 and 6 and 7 175

9 limit 8 to (english language and yr="2000 - 2024") 154

10 limit 9 to "remove medline records" 32

**CINAHL**

(TI ("nutrition* outcome*" or "nutrition* status" or "diet* diversi*" or micronutrient* or anthropom* or food* or macronutrient* or nutrition* or "food consumption*" or diet* or "food secur*" or "food insecur*" or "food security governance" or "governance for food security")) AND (TI (“Sub-saharan Africa” OR “Central Africa” OR “Eastern Africa” OR “Southern Africa” OR “Western Africa” OR Angola OR Benin OR Botswana OR Burkina Faso OR Burundi OR “Cabo Verde” OR Cameroon OR “Central African Republic” OR Chad OR Comoros OR Congo OR “Democratic Republic of Congo” OR “Republic of congo” OR “Cote d'Ivoire” OR “Equatorial Guinea” OR Eritrea OR Eswatini OR Swaziland OR Ethiopia OR Gabon OR Gambia OR Ghana OR Guinea OR Guinea-Bissau OR Kenya OR Lesotho OR Liberia OR Madagascar OR Malawi OR Mali OR Mauritania OR Mauritius OR Mozambique OR Namibia OR Niger OR Nigeria OR Rwanda OR “Sao Tome and Principe” OR Senegal OR Seychelles OR Sierra Leone OR Somalia OR “South Africa” OR “South Sudan” OR Sudan OR Tanzania OR Togo OR Uganda OR Zambia OR Zimbabwe)) AND (TI ("land tenure" or "property right*" or "land right*" or "property right*" or "land property right" or "land tenure security" or regulation or "land valuation" or "land taxation" or "land size" or "land access" or "documented legal right*" or "de facto land possession" or "land registration" or "land certification" or "land tenure regulari*" or "land reform" or "customary land tenure" or "land governance" or conversion or freehold* or titl* or codification or recognition or customary or certification or right* or "farmers' right*" or "land expansion" or "land registration" or "land information") OR TI ("value chain" or value-chain or "value crop*" or "value-crop*" or "market access" or harvesting or storage or "waste disposal" or "food storage" or "grain storage silo" or "value chain development" or "food processing cost" or "agriculture cooperatives" or "marketing cooperatives" or "farmer cooperative" or "agricultural marketing cooperative" or "agriculture coop" or "agri-coop" or "marketing coop" or "agriculture co-op" or "cooperative societies" or subsid* or "input support*" or "credit support*" or "agricultural technical support" or "improved market access" or "food processing" or "diversified income sources" or "distance to market" or extension or "organisation membership*" or "agricultural commercialization" or "livelihood system" or "market access" or "farm to market transport infrastructure" or "output market information" or "transport network*" or "food quality" or "food spoilage" or "marketing cost*" or "transport cost*" or "market information" or "food price*" or "market trade*" or "market demand*" or "export transport" or "domestic transport" or transport* or road* or bridge* or waterway* or boat* or rail* or "train tracks" or ports or airport* or "border crossing*" or "distribution facilit*" or highway* or distribut* or freight or access* or connect* or rural or electric* or power or transmission or grid or energy or "information dissemination" or "communication technolog*" or "information technolog*" or "information service*" or "social network*" or "peer network*" or "commodity exchange" or market* or trader* or "processor*" or "agro-exporter*" or "agroexporter*" or "dealer*" or "agro-dealer*" or "agrodealer*" or "wholesaler*" or "miller*" or "village merchant*" or "commission agent*" or "broker*" or "retailer*" or "aggregator*" or "buyer*" or "trading firm*" or "farm-firm*" or "tender*" or "service provider*" or "trucker*" or "transporter*" or "logistics enterprise*" or "third-party logistics" or "freight forwarder*" or "food manufacturer*" or "packager*" or "distributor* commit*" or contract* or "agreement* fixed pric*" or "arrang*" or "guarant*" or "innovative marketing channel*" or "new marketing channel*" or commercialization or commercialisation or "high-value chain*" or "high-value market*" or "high value agricultur*" or "agrifood transformation*" or "agri-food transformation*" or "e-commerce" or "market reform*" or "market linkage*" or "online agricultural platform*" or "agri-platform*" or agriplatform* or "market hub*" or "internet kiosk*" or storage or waste or "waste management" or preservation or "storage device" or "preservation technique" or "post-harvest loss" or "income generation" or "bio energy" or bio-energy or "contract farming" or "food production" or "Crop cultivation" or livestock raising or aquaculture or "Harvesting" or "Timing of harvest" or "harvesting technique*" or drying or cleaning or Heating or Canning or Drying or dehydration or Juicing or Fermentation or Freezing or "Modified atmosphere packaging" or Pasteurisation or Smoking or Additives or "Pulsed electric fields technology" or "pickling of sugaring" or "Post-Harvest Handling" or Sorting or grading or packaging or storage or vacuuming or milling or Distribution or Transportation or logistics or "supply chain" or management or "Marketing and Retail" or "Wholesale markets" or supermarkets or "farmers' markets" or "online platforms" or "Consumption" or "Food preparation" or cooking or "consumption by end-users") OR TI ("Agricultural marketing" or "marketing regulation" or "agricultural marketing law*" or "agriculture regulation" or "agriculture legislation" or "food standards" or "food regulation" or "food safety" or "food safety standard*" or "food grading" or "food price" or "food price regulation" or "term of trade" or "trading practice*" or condition or trade or "unfair trade" or "unfair trade practice*" or "price control*" or "export support*" or "tax concession*" or quota* or tariff* or "import control*" or "crop regulation" or "price support*" or "crop control*" or "market regulation")) AND (TI ("land tenure" or "property right*" or "land right*" or "property right*" or "land property right" or "land tenure security" or regulation or "land valuation" or "land taxation" or "land size" or "land access" or "documented legal right*" or "de facto land possession" or "land registration" or "land certification" or "land tenure regulari*" or "land reform" or "customary land tenure" or "land governance" or conversion or freehold* or titl* or codification or recognition or customary or certification or right* or "farmers' right*" or "land expansion" or "land registration" or "land information"))

**ProQuest**

title(program* OR polic* OR strateg* OR legislation* OR law* OR intervention* OR technique* OR planning OR practice* OR fiscal OR regulation* OR sustainable OR tax* OR subsid* OR procurement* OR incentive* OR Impact* OR effect* OR evaluat* OR impact evaluat* impact assessment OR assess OR assessing OR assessment OR analyze OR analyse OR analyzing OR analysing OR analysis OR analytical OR estimate OR estimating OR estimation OR cause OR causal OR impact* OR evaluat* OR effect* OR experiment* OR trial OR random* OR quasi* OR natural experiment* OR discontinuity OR fixed effect* OR regression OR difference in differences OR instrumental variable* OR matching OR inverse propensity weight) AND title("land tenure" OR "property right*" OR "land right*" OR "property right*" OR "land property right" OR "land tenure security" OR regulation OR "land valuation" OR "land taxation" OR "land size" OR "land access" OR "documented legal right*" OR "de facto land possession" OR "land registration" OR "land certification" OR "land tenure regulari*" OR "land reform" OR "customary land tenure" OR "land governance" OR conversion OR freehold* OR titl* OR codification OR recognition OR customary OR certification OR right* OR "farmers' right*" OR "land expansion" OR "land registration" OR "land information" OR "value chain" OR value-chain OR "value crop*" OR "value-crop*" OR "market access" OR harvesting OR storage OR "waste disposal" OR "food storage" OR "grain storage silo" OR "value chain development" OR "food processing cost" OR "agriculture cooperatives" OR "marketing cooperatives" OR "farmer cooperative" OR "agricultural marketing cooperative" OR "agriculture coop" OR "agri-coop" OR "marketing coop" OR "agriculture co-op" OR "cooperative societies" OR subsid* OR "input support*" OR "credit support*" OR "agricultural technical support" OR "improved market access" OR "food processing" OR "diversified income sources" OR "distance to market" OR extension OR "organisation membership*" OR "agricultural commercialization" OR "livelihood system" OR "market access" OR "farm to market transport infrastructure" OR "output market information" OR "transport network*" OR "food quality" OR "food spoilage" OR "marketing cost*" OR "transport cost*" OR "market information" OR "food price*" OR "market trade*" OR "market demand*" OR "export transport" OR "domestic transport" OR transport* OR road* OR bridge* OR waterway* OR boat* OR rail* OR "train tracks" OR ports OR airport* OR "border crossing*" OR "distribution facilit*" OR highway* OR distribut* OR freight OR access* OR connect* OR rural OR electric* OR power OR transmission OR grid OR energy OR "information dissemination" OR "communication technolog*" OR "information technolog*" OR "information service*" OR "social network*" OR "peer network*" OR "commodity exchange" OR market* OR trader* OR "processor*" OR "agro-exporter*" OR "agroexporter*" OR "dealer*" OR "agro-dealer*" OR "agrodealer*" OR "wholesaler*" OR "miller*" OR "village merchant*" OR "commission agent*" OR "broker*" OR "retailer*" OR "aggregator*" OR "buyer*" OR "trading firm*" OR "farm-firm*" OR "tender*" OR "service provider*" OR "trucker*" OR "transporter*" OR "logistics enterprise*" OR "third-party logistics" OR "freight forwarder*" OR "food manufacturer*" OR "packager*" OR "distributor* commit*" OR contract* OR "agreement* fixed pric*" OR "arrang*" OR "guarant*" OR "innovative marketing channel*" OR "new marketing channel*" OR commercialization OR commercialisation OR "high-value chain*" OR "high-value market*" OR "high value agricultur*" OR "agrifood transformation*" OR "agri-food transformation*" OR "e-commerce" OR "market reform*" OR "market linkage*" OR "online agricultural platform*" OR "agri-platform*" OR agriplatform* OR "market hub*" OR "internet kiosk*" OR storage OR waste OR "waste management" OR preservation OR "storage device" OR "preservation technique" OR "post-harvest loss" OR "income generation" OR "bio energy" OR bio-energy OR "contract farming" OR "Agricultural marketing" OR "marketing regulation" OR "agricultural marketing law*" OR "agriculture regulation" OR "agriculture legislation" OR "food standards" OR "food regulation" OR "food safety" OR "food safety standard*" OR "food grading" OR "food price" OR "food price regulation" OR "term of trade" OR "trading practice*" OR condition OR trade OR "unfair trade" OR "unfair trade practice*" OR "price control*" OR "export support*" OR "tax concession*" OR quota* OR tariff* OR "import control*" OR "crop regulation" OR "price support*" OR "crop control*" OR "market regulation") AND title("nutrition* outcome*" OR "nutrition* status" OR "diet* diversi*" OR micronutrient* OR anthropom* OR food* OR macronutrient* OR nutrition* OR "food consumption*" OR diet* OR "food secur*" OR "food insecur*" OR "food security governance" OR "governance for food security") AND title(Sub-saharan Africa OR Central Africa OR Eastern Africa OR Southern Africa OR Western Africa OR Angola OR Benin OR Botswana OR Burkina Faso OR Burundi OR Cabo Verde OR Cameroon OR Central African Republic OR Chad OR Comoros OR Congo OR Democratic Republic of Congo OR Republic of congo OR Cote d'Ivoire OR Equatorial Guinea OR Eritrea OR Eswatini OR Swaziland OR Ethiopia OR Gabon OR Gambia OR Ghana OR Guinea OR Guinea-Bissau OR Kenya OR Lesotho OR Liberia OR Madagascar OR Malawi OR Mali OR Mauritania OR Mauritius OR Mozambique OR Namibia OR Niger OR Nigeria OR Rwanda OR "Sao Tome and Principe" OR Senegal OR Seychelles OR Sierra Leone OR Somalia OR South Africa OR South Sudan OR Sudan OR Tanzania OR Togo OR Uganda OR Zambia OR Zimbabwe) AND stype.exact("Conference Papers & Proceedings" OR "Books" OR "Scholarly Journals") AND at.exact("Book Chapter" OR "Annual Report" OR "Working Paper/Pre-Print" OR "Correction/Retraction" OR "Conference Paper" OR "Book" OR "Correspondence" OR "Conference" OR "Technical Report" OR "Bibliography" OR "Statistics/Data Report" OR "Conference Proceeding" OR "Article") AND la.exact("English") AND PEER(yes) AND stype.exact("Conference Papers & Proceedings" OR "Scholarly Journals") AND at.exact("Annual Report" OR "Bibliography" OR "Statistics/Data Report" OR "Working Paper/Pre-Print" OR "Conference Proceeding" OR "Correction/Retraction" OR "Conference Paper" OR "Correspondence" OR "Conference" OR "Technical Report" OR "Article")

**Web of Science**

program* or polic* or strateg* or legislation* or law* or intervention* or technique* or planning or practice* or fiscal or regulation* or sustainable or tax* or subsid* or procurement* or incentive* or Impact* or effect* or evaluat* or impact evaluat* impact assessment or assess or assessing or assessment or analyze or analyse or analyzing or analysing or analysis or analytical or estimate or estimating or estimation or cause or causal or impact* or evaluat* or effect* or experiment* or trial or random* or quasi* or natural experiment* or discontinuity or fixed effect* or regression or difference in differences or instrumental variable* or matching or inverse propensity weight (Title) and "value chain" or value-chain or "value crop*" or "value-crop*" or "market access" or harvesting or storage or "waste disposal" or "food storage" or "grain storage silo" or "value chain development" or "food processing cost" or "agriculture cooperatives" or "marketing cooperatives" or "farmer cooperative" or "agricultural marketing cooperative" or "agriculture coop" or "agri-coop" or "marketing coop" or "agriculture co-op" or "cooperative societies" or subsid* or "input support*" or "credit support*" or "agricultural technical support" or "improved market access" or "food processing" or "diversified income sources" or "distance to market" or extension or "organisation membership*" or "agricultural commercialization" or "livelihood system" or "market access" or "farm to market transport infrastructure" or "output market information" or "transport network*" or "food quality" or "food spoilage" or "marketing cost*" or "transport cost*" or "market information" or "food price*" or "market trade*" or "market demand*" or "export transport" or "domestic transport" or transport* or road* or bridge* or waterway* or boat* or rail* or "train tracks" or ports or airport* or "border crossing*" or "distribution facilit*" or highway* or distribut* or freight or access* or connect* or rural or electric* or power or transmission or grid or energy or "information dissemination" or "communication technolog*" or "information technolog*" or "information service*" or "social network*" or "peer network*" or "commodity exchange" or market* or trader* or "processor*" or "agro-exporter*" or "agroexporter*" or "dealer*" or "agro-dealer*" or "agrodealer*" or "wholesaler*" or "miller*" or "village merchant*" or "commission agent*" or "broker*" or "retailer*" or "aggregator*" or "buyer*" or "trading firm*" or "farm-firm*" or "tender*" or "service provider*" or "trucker*" or "transporter*" or "logistics enterprise*" or "third-party logistics" or "freight forwarder*" or "food manufacturer*" or "packager*" or "distributor* commit*" or contract* or "agreement* fixed pric*" or "arrang*" or "guarant*" or "innovative marketing channel*" or "new marketing channel*" or commercialization or commercialisation or "high-value chain*" or "high-value market*" or "high value agricultur*" or "agrifood transformation*" or "agri-food transformation*" or "e-commerce" or "market reform*" or "market linkage*" or "online agricultural platform*" or "agri-platform*" or agriplatform* or "market hub*" or "internet kiosk*" or storage or waste or "waste management" or preservation or "storage device" or "preservation technique" or "post-harvest loss" or "income generation" or "bio energy" or bio-energy or "contract farming" or "food production" or "Crop cultivation" or livestock raising or aquaculture or "Harvesting" or "Timing of harvest" or "harvesting technique*" or drying or cleaning or Heating or Canning or Drying or dehydration or joining or Fermentation or Freezing or "Modified atmosphere packaging" or Pasteurisation or Smoking or Additives or "Pulsed electric fields technology" or "pickling of sugaring" or "Post-Harvest Handling" or Sorting or grading or packaging or storage or vacuming or milling or Distribution or Transportation or logistics or "supply chain" or management or "Marketing and Retail" or "Wholesale markets" or supermarkets or "farmers' markets" or "online platforms" or "Consumption" or "Food preparation" or cooking or "consumption by end-users" (Title) and "nutrition* outcome*" or "nutrition* status" or "diet* diversi*" or micronutrient* or anthropom* or food* or macronutrient* or nutrition* or "food consumption*" or diet* or "food secur*" or "food insecur*" or "food security governance" or "governance for food security" (Title) and Sub-saharan Africa or Central Africa or Eastern Africa or Southern Africa or Western Africa or Angola or Benin or Botswana or Burkina Faso or Burundi or Cabo Verde or Cameroon or Central African Republic or Chad or Comoros or Congo or Democratic Republic of Congo or Republic of congo or Cote d'Ivoire or Equatorial Guinea or Eritrea or Eswatini or Swaziland or Ethiopia or Gabon or Gambia or Ghana or Guinea or Guinea-Bissau or Kenya or Lesotho or Liberia or Madagascar or Malawi or Mali or Mauritania or Mauritius or Mozambique or Namibia or Niger or Nigeria or Rwanda or “Sao Tome and Principe” or Senegal or Seychelles or Sierra Leone or Somalia or South Africa or South Sudan or Sudan or Tanzania or Togo or Uganda or Zambia or Zimbabwe (Title) and Article or Proceeding Paper (Document Types)

**Scopus**

TITLE-ABS-KEY ( ( program* OR polic* OR strateg* OR legislation* OR law* OR intervention* OR technique* OR planning OR practice* OR fiscal OR regulation* OR sustainable OR tax* OR subsid* OR procurement* OR incentive* OR impact* OR effect* OR evaluat* OR "impact evaluat*" OR "impact assessment" OR assess OR assessing OR assessment OR analyze OR analyse OR analyzing OR analysing OR analysis OR analytical OR estimate OR estimating OR estimation OR cause OR causal OR impact* OR evaluat* OR effect* OR experiment* OR trial OR random* OR quasi* OR "natural experiment*" OR discontinuity OR fixed AND effect* OR regression OR "difference in differences" OR "instrumental variable*" OR matching OR "inverse propensity weight" ) AND ( "land tenure" OR "property right*" OR "land right*" OR "property right*" OR "land property right" OR "land tenure security" OR regulation OR "land valuation" OR "land taxation" OR "land size" OR "land access" OR "documented legal right*" OR "de facto land possession" OR "land registration" OR "land certification" OR "land tenure regulari*" OR "land reform" OR "customary land tenure" OR "land governance" OR conversion OR freehold* OR titl* OR codification OR recognition OR customary OR certification OR right* OR "farmers' right*" OR "land expansion" OR "land registration" OR "land information" OR "value chain" OR value-chain OR "value crop*" OR "value-crop*" OR "market access" OR harvesting OR storage OR "waste disposal" OR "food storage" OR "grain storage silo" OR "value chain development" OR "food processing cost" OR "agriculture cooperatives" OR "marketing cooperatives" OR "farmer cooperative" OR "agricultural marketing cooperative" OR "agriculture coop" OR "agri-coop" OR "marketing coop" OR "agriculture co-op" OR "cooperative societies" OR subsid* OR "input support*" OR "credit support*" OR "agricultural technical support" OR "improved market access" OR "food processing" OR "diversified income sources" OR "distance to market" OR extension OR "organisation membership*" OR "agricultural commercialization" OR "livelihood system" OR "market access" OR "farm to market transport infrastructure" OR "output market information" OR "transport network*" OR "food quality" OR "food spoilage" OR "marketing cost*" OR "transport cost*" OR "market information" OR "food price*" OR "market trade*" OR "market demand*" OR "export transport" OR "domestic transport" OR transport* OR road* OR bridge* OR waterway* OR boat* OR rail* OR "train tracks" OR ports OR airport* OR "border crossing*" OR "distribution facilit*" OR highway* OR distribut* OR freight OR access* OR connect* OR rural OR electric* OR power OR transmission OR grid OR energy OR "information dissemination" OR "communication technolog*" OR "information technolog*" OR "information service*" OR "social network*" OR "peer network*" OR "commodity exchange" OR market* OR trader* OR "processor*" OR "agro-exporter*" OR "agroexporter*" OR "dealer*" OR "agro-dealer*" OR "agrodealer*" OR "wholesaler*" OR "miller*" OR "village merchant*" OR "commission agent*" OR "broker*" OR "retailer*" OR "aggregator*" OR "buyer*" OR "trading firm*" OR "farm-firm*" OR "tender*" OR "service provider*" OR "trucker*" OR "transporter*" OR "logistics enterprise*" OR "third-party logistics" OR "freight forwarder*" OR "food manufacturer*" OR "packager*" OR "distributor* commit*" OR contract* OR "agreement* fixed pric*" OR "arrang*" OR "guarant*" OR "innovative marketing channel*" OR "new marketing channel*" OR commercialization OR commercialisation OR "high-value chain*" OR "high-value market*" OR "high value agricultur*" OR "agrifood transformation*" OR "agri-food transformation*" OR "e-commerce" OR "market reform*" OR "market linkage*" OR "online agricultural platform*" OR "agri-platform*" OR agriplatform* OR "market hub*" OR "internet kiosk*" OR storage OR waste OR "waste management" OR preservation OR "storage device" OR "preservation technique" OR "post-harvest loss" OR "income generation" OR "bio energy" OR bio-energy OR "contract farming" OR "food production" OR "Crop cultivation" OR "livestock raising" OR aquaculture OR "Harvesting" OR "Timing of harvest" OR "harvesting technique*" OR drying OR cleaning OR heating OR canning OR drying OR dehydration OR juicing OR fermentation OR freezing OR "Modified atmosphere packaging" OR pasteurisation OR smoking OR additives OR "Pulsed electric fields technology" OR "pickling of sugaring" OR "Post-Harvest Handling" OR sorting OR grading OR packaging OR storage OR vacuuming OR milling OR distribution OR transportation OR logistics OR "supply chain" OR management OR "Marketing and Retail" OR "Wholesale markets" OR supermarkets OR "farmers' markets" OR "online platforms" OR "Consumption" OR "Food preparation" OR cooking OR "consumption by end-users" OR "Agricultural marketing" OR "marketing regulation" OR "agricultural marketing law*" OR "agriculture regulation" OR "agriculture legislation" OR "food standards" OR "food regulation" OR "food safety" OR "food safety standard*" OR "food grading" OR "food price" OR "food price regulation" OR "term of trade" OR "trading practice*" OR condition OR trade OR "unfair trade" OR "unfair trade practice*" OR "price control*" OR "export support*" OR "tax concession*" OR quota* OR tariff* OR "import control*" OR "crop regulation" OR "price support*" OR "crop control*" OR "market regulation" ) AND ( "nutrition* outcome*" OR "nutrition* status" OR "diet* diversi*" OR micronutrient* OR anthropom* OR food* OR macronutrient* OR nutrition* OR "food consumption*" OR diet* OR "food secur*" OR "food insecur*" OR "food security governance" OR "governance for food security" ) AND ( "Sub-saharan Africa" OR "Central Africa" OR "Eastern Africa" OR "Southern Africa" OR "Western Africa" OR angola OR benin OR botswana OR burkina AND faso OR burundi OR "Cabo Verde" OR cameroon OR "Central African Republic" OR chad OR comoros OR congo OR "Democratic Republic of Congo" OR "Republic of congo" OR "Cote d'Ivoire" OR "Equatorial Guinea" OR eritrea OR eswatini OR swaziland OR ethiopia OR gabon OR gambia OR ghana OR guinea OR guinea-bissau OR kenya OR lesotho OR liberia OR madagascar OR malawi OR mali OR mauritania OR mauritius OR mozambique OR namibia OR niger OR nigeria OR rwanda OR "Sao Tome and Principe" OR senegal OR seychelles OR sierra AND leone OR somalia OR "South Africa" OR "South Sudan" OR sudan OR tanzania OR togo OR uganda OR zambia OR zimbabwe ) )

**Cochrane**

 (program* or polic* or strateg* or legislation* or law* or intervention* or technique* or planning or practice* or fiscal or regulation* or sustainable or tax* or subsid* or procurement* or incentive* or Impact* or effect* or evaluat* or impact evaluat* impact assessment or assess or assessing or assessment or analyze or analyse or analyzing or analysing or analysis or analytical or estimate or estimating or estimation or cause or causal or impact* or evaluat* or effect* or experiment* or trial or random* or quasi* or natural experiment* or discontinuity or fixed effect* or regression or difference in differences or instrumental variable* or matching or inverse propensity weight):ti AND (“value chain” OR value-chain OR “value crop*” OR “value-crop*” or “market access” or harvesting or storage or “waste disposal” or “food storage” or “grain storage silo” or “value chain development” or “food processing cost” or “agriculture cooperatives” OR “marketing cooperatives” OR “farmer cooperative” OR “agricultural marketing cooperative” OR “agriculture coop” OR “agri-coop” OR “marketing coop” OR “agriculture co-op” OR “cooperative societies” or subsid* OR “input support*” OR “credit support*” or “agricultural technical support” or “improved market access” or “food processing” or “diversified income sources” or “distance to market” or extension or “organisation membership*” or “agricultural commercialization” or “livelihood system” or “market access” or “farm to market transport infrastructure” or “output market information” or “transport network*” or “food quality” or “food spoilage” or “marketing cost*” or “transport cost*” or “market information” or “food price*” or “market trade*” or “market demand*” or “export transport” or “domestic transport” or transport* OR road* OR bridge* OR waterway* OR boat* OR rail* OR "train tracks" OR ports OR airport* OR "border crossing*" OR "distribution facilit*" OR highway* OR distribut* OR freight OR access* OR connect* OR rural OR electric* OR power OR transmission OR grid OR energy or “information dissemination” OR "communication technolog*" OR "information technolog*" OR “information service*” OR "social network*" OR "peer network*" or “commodity exchange” OR market* OR trader* OR "processor*" OR "agro-exporter*" OR "agroexporter*" OR "dealer*" OR "agro-dealer*" OR "agrodealer*" OR "wholesaler*" OR "miller*" OR "village merchant*" OR "commission agent*" OR "broker*" OR "retailer*" OR "aggregator*" OR "buyer*" OR "trading firm*" OR "farm-firm*" OR "tender*" OR "service provider*" OR "trucker*" OR "transporter*" OR "logistics enterprise*" OR "third-party logistics" OR "freight forwarder*" OR "food manufacturer*" OR "packager*" OR “distributor* commit*” OR contract* OR agreement* "fixed pric*" OR "arrang*" OR "guarant*" OR "innovative marketing channel*" OR "new marketing channel*" OR commercialization OR commercialisation OR "high-value chain*" OR "high-value market*" OR "high value agricultur*" OR "agrifood transformation*" OR "agri-food transformation*" OR "e-commerce" OR "market reform*" OR "market linkage*" OR "online agricultural platform*" OR "agri-platform*" OR agriplatform* OR "market hub*" OR "internet kiosk*" or “land tenure” or “property right*” OR “land right*” or “property right*” or “land property right” or “land tenure security” or regulation or “land valuation” or “land taxation” or “land size” or “land access” or “documented legal right*” or “de facto land possession” or “land registration” OR “land certification” OR “land tenure regulari*” OR “land reform” or “customary land tenure” OR “land governance” or conversion or freehold* or titl* or codification or recognition or customary or certification or right* or “farmers’ right*” or “land expansion” or “land registration” or “land information” or "Agricultural marketing" or "marketing regulation" or "agricultural marketing law*" or "agriculture regulation" or "agriculture legislation" or "food standards" or "food regulation" or "food safety" or "food safety standard*" or "food grading" or "food price" or "food price regulation" or "term of trade" or "trading practice*" or condition or trade or "unfair trade" or "unfair trade practice*" or "price control*" or "export support*" or "tax concession*" or quota* or tariff* or "import control*" or "crop regulation" or "price support*" or "crop control*" or "market regulation"):ti AND (Sub-saharan Africa or Central Africa or Eastern Africa or Southern Africa or Western Africa or Angola or Benin or Botswana or Burkina Faso or Burundi or Cabo Verde or Cameroon or Central African Republic or Chad or Comoros or Congo or Democratic Republic of Congo or Republic of congo or Cote d'Ivoire or Equatorial Guinea or Eritrea or Eswatini or Swaziland or Ethiopia or Gabon or Gambia or Ghana or Guinea or Guinea-Bissau or Kenya or Lesotho or Liberia or Madagascar or Malawi or Mali or Mauritania or Mauritius or Mozambique or Namibia or Niger or Nigeria or Rwanda or “Sao Tome and Principe” or Senegal or Seychelles or Sierra Leone or Somalia or South Africa or South Sudan or Sudan or Tanzania or Togo or Uganda or Zambia or Zimbabwe):ti AND ("nutrition* outcome*" or "nutrition* status" or "diet* diversi*" or micronutrient* or anthropom* or food* or macronutrient* or nutrition* or "food consumption*" or diet* or "food secur*" or "food insecur*" or "food security governance" or "governance for food security"):ti (Word variations have been searched)" with Cochrane Library publication date Between Jan 2000 and Dec 2023, in Cochrane Reviews, Trials (Word variations have been searched)
