# Supplementary material for: The Effect of Food Value Chain Interventions on Food Security in Sub‐Saharan Africa: A Systematic Review and Meta‐Analysis
Source: Food Sci Nutr. 2026 Jun 15;14(6):e71881. doi: 10.1002/fsn3.71881 (PMC13269678; doi:10.1002/fsn3.71881)
Supplement: Supplementary file 3 — Table S2: fsn371881‐sup‐0003‐TableS2.docx. [file FSN3-14-e71881-s001.docx]

**Supplementary Table 2**

**Field product testing: Studies that tested agricultural products such as seed varieties, pesticides, fertilizers, storage materials, or bio-stimulants, for the purpose of establishing efficacy, performance, or safety**

1. Kotu, B.H., Abass, A.B., Hoeschle-Zeledon, I., Mbwambo, H. and Bekunda, M., 2019. Exploring the profitability of improved storage technologies and their potential impacts on food security and income of smallholder farm households in Tanzania. Journal of Stored Products Research, 82, pp.98-109.
2. Williams SB, Baributsa D, Woloshuk C. Assessing Purdue Improved Crop Storage (PICS) bags to mitigate fungal growth and aflatoxin contamination. Journal of stored products research. 2014 Oct 1;59:190-6.
3. Baoua IB, Amadou L, Bakoye O, Baributsa D, Murdock LL. Triple bagging hermetic technology for post-harvest preservation of paddy rice Oryza sativa L. in the Sahel of West Africa. Journal of Stored Products Research. 2016 Jul 1;68:73-9.

**Modelling**

1. Ntiamoah EB, Li D, Appiah-Otoo I, Twumasi MA, Yeboah EN. Towards a sustainable food production: modelling the impacts of climate change on maize and soybean production in Ghana. Environmental Science and Pollution Research. 2022 Oct;29(48):72777-96.
2. Kinkpe, Agossoussi Thierry, Jonas Luckmann, and Harald Grethe. "Welfare effects of food-processing development in agriculture-based economies: a CGE analysis for Benin." Applied Economics 56.57 (2024): 7825-7844.
3. Fagbemi, F., Oke, D.F. and Fajingbesi, A., 2023. Climate-resilient development: An approach to sustainable food production in sub-Saharan Africa. Future Foods, 7, p.100216
4. Tette, A. S. K., Odey, G., Ahmad, M. J., Adelodun, B., & Choi, K. S. (2024). Crop Water Use and a Gravity Model Exploration of Virtual Water Trade in Ghana’s Cereal Agriculture. Water, 16(15), 2077.
5. Tetteh, B., Baidoo, S. T., & Takyi, P. O. (2022). The effects of climate change on food production in Ghana: evidence from Maki (2012) cointegration and frequency domain causality models. Cogent Food & Agriculture, 8(1), 2111061.

**Reviews**

1. Donovan J, Gelli A. Designing interventions in local value chains for improved health and nutrition: Insights from Malawi. World Development Perspectives. 2019 Dec 1;16:100149.

**Ineligible / Correlational / Not directly FVC-focused**

1. Anteneh M. Climate variability patterns and farmers’ perceptions of its impact on food production: A case study of the Gelda watershed in the Lake Tana Basin in Northwest Ethiopia. Air, Soil and Water Research. 2022 Nov;15:11786221221135093.
2. Atara, A., Tolossa, D., & Denu, B. (2020). Analysis of rural households’ resilience to food insecurity: Does livelihood systems/choice/matter? The case of Boricha woreda of sidama zone in southern Ethiopia. Environmental Development, 35, 100530.
3. Barasa, B., Watsusi, C.W., Gudoyi, P.M., Lutaaya, N., Gumisiriza, L.T. and Magaya, J.P., 2023. Desert locust invasion in Uganda: effects on household food consumption and effective control interventions. Sustainability, 15(19), p.14496.
4. Behrendt, L., Kolossa, S., Vrachioli, M., Abate Kassa, G., Ayenew, H., Gedrich, K., Crinot, G.F., Houssou, P. and Sauer, J., 2024. Assessing the impact of a personalised application-based nutrition intervention on carbohydrate intake in rural Benin. Agriculture & Food Security, 13(1), p.15.
5. Bürkert A, Piepho HP, Bationo A. Multi-site time-trend analysis of soil fertility management effects on crop production in sub-Saharan West Africa. Experimental Agriculture. 2002 Apr;38(2):163-83.
6. Dinssa, F.F., Minja, R., Kariuki, T., Mbwambo, O., Schafleitner, R. and Hanson, P., 2022. Gender-disaggregated farmers participatory variety selection in amaranth multilocation trials in Kenya and Tanzania. HortTechnology, 32(3), pp.288-303.
7. Nkomoki, W., Bavorová, M. and Banout, J., 2018. Adoption of sustainable agricultural practices and food security threats: Effects of land tenure in Zambia. Land use policy, 78, pp.532-538.
8. Fang T, Tran A, Oranga B, Kopper R, Kang Y. Associations between a cash voucher intervention, food consumption, and coping strategies in Somali food-insecure populations. Agriculture & Food Security. 2024 Feb 7;13(1):8.
9. Feyisa M. The Effect of Productive Safety Net Programme on Household Food Consumption and Dietary Diversity in Ethiopia.
10. Graef F, Schneider I, Fasse A, Germer JU, Gevorgyan E, Haule F, Hoffmann H, Kahimba FC, Kashaga L, Kissoly L, Lambert C. Assessment of upgrading strategies to improve regional food systems in Tanzania: Food processing, waste management and bioenergy, and income generation. Outlook on AGRICULTURE. 2015 Sep;44(3):179-86.
11. Habtu M, Agena AG, Umugwaneza M, Mochama M, Munyanshongore C. Effect of integrated nutrition‐sensitive and nutrition‐specific intervention package on maternal malnutrition among pregnant women in Rwanda. Maternal & Child Nutrition. 2022 Jul;18(3):e13367.
12. Handa S, Otchere F, Sirma P, Evaluation Study Team. More evidence on the impact of government social protection in sub‐Saharan Africa: Ghana, Malawi, and Zimbabwe. Development Policy Review. 2022 May;40(3):e12576.
13. Hetherington JB, Wiethoelter AK, Negin J, Mor SM. Livestock ownership, animal source foods and child nutritional outcomes in seven rural village clusters in Sub-Saharan Africa. Agriculture & Food Security. 2017 Jan 24;6(1):9.
14. Khonje MG, Ricker-Gilbert J, Muyanga M, Qaim M. Farm-level production diversity and child and adolescent nutrition in rural sub-Saharan Africa: a multicountry, longitudinal study. The Lancet Planetary Health. 2022 May 1;6(5):e391-9.
15. Mengistu DD, Degaga DT, Tsehay AS. Analyzing the contribution of crop diversification in improving household food security among wheat dominated rural households in Sinana District, Bale Zone, Ethiopia. Agriculture & Food Security. 2021 Feb 28;10(1):7.
16. Mensah, Clement, and Abdulrazak Karriem. "Harnessing public food procurement for sustainable rural livelihoods in South Africa through the National School Nutrition Programme: a qualitative assessment of contributions and challenges." Sustainability 13, no. 24 (2021): 13838.
17. Mkwambisi DD, Fraser ED, Dougill AJ. Urban agriculture and poverty reduction: Evaluating how food production in cities contributes to food security, employment and income in Malawi. Journal of International Development. 2011 Mar;23(2):181-203.
18. Mosites, Emily M., Peter M. Rabinowitz, Samuel M. Thumbi, Joel M. Montgomery, Guy H. Palmer, Susanne May, Ali Rowhani-Rahbar, Marian L. Neuhouser, and Judd L. Walson. "The relationship between livestock ownership and child stunting in three countries in Eastern Africa using national survey data." PloS one 10, no. 9 (2015): e0136686.
19. Mponela, P., Manda, J., Kinyua, M., & Kihara, J. (2023). The impact of participatory action research and endogenous integrated soil fertility management on farm-gate dietary outputs in northern Tanzania. Heliyon, 9(11).
20. Murage AW, Midega CA, Pittchar JO, Pickett JA, Khan ZR. Determinants of adoption of climate-smart push-pull technology for enhanced food security through integrated pest management in eastern Africa. Food Security. 2015 Jun;7(3):709-24.
21. Nkomoki W, Bavorová M, Banout J. Adoption of sustainable agricultural practices and food security threats: Effects of land tenure in Zambia. Land use policy. 2018 Nov 1;78:532-8.
22. Obayelu, A.E., Okuneye, P.A., Shittu, A.M., Afolami, C.A. and Dipeolu, A.O., 2016. Determinants and the perceived effects of adoption of sustainable improved food crop technologies by smallholder farmers along the value chain in Nigeria. Journal of Agriculture and Environment for International Development (JAEID), 110(1), pp.155-172.
23. Onono, Maricianah A., Gladys Odhiambo, Lila Sheira, Amy Conroy, Torsten B. Neilands, Elizabeth A. Bukusi, and Sheri D. Weiser. "The role of food security in increasing adolescent girls’ agency towards sexual risk taking: qualitative findings from an income generating agricultural intervention in southwestern Kenya." BMC public health 21, no. 1 (2021): 2028.
24. Pedrero-Tomé, R., Marrodán, M. D., López-Ejeda, N., Escruela, M., Rocaspana, M., Vargas, A., ... & Lanusse, C. (2023). Impact of integrated preventive and curative health package on nutritional status of children under 2 years of age in the health area of Tama, Tahoua region (Niger). Frontiers in Nutrition, 10, 1259706.
25. Rigolot, Cyrille, P. De Voil, Sabine Douxchamps, D. Prestwidge, Mark Van Wijk, Philip K. Thornton, Daniel Rodriguez, B. Henderson, D. Medina, and Mario Herrero. "Interactions between intervention packages, climatic risk, climate change and food security in mixed crop–livestock systems in Burkina Faso." Agricultural Systems 151 (2017): 217-224.
26. Sarr M, Majili Z, Khalili N, Matavel CE, Mbwana HA, Kaingo J, Löhr K, Rybak C. Adoption of processing technologies and innovative food preservation techniques: findings from smallholders in the Lindi Region in Tanzania. Frontiers in Sustainable Food Systems. 2024 Jan 5;7:1169578.
27. Sebotsa ML, Lues L. An Evaluation of the Implementation and Management of the Strategies adopted by the Government to Improve Food Security in Lesotho. Journal for New Generation Sciences. 2011 Jan 1;9(2):43-55.
28. Orou Seko M, Ossebi W, Houngbedji CA, Kreppel K, Dao D, Bonfoh B. Effectiveness and cost of an incentive-based intervention on food safety and income in “dibiteries” in Dakar, Senegal. BMC Public Health. 2022 Mar 11;22(1):484.
29. Sherr L, Roberts KJ, Tomlinson M, Skeen S, Mebrahtu H, Gordon S, du Toit S, Haag K, Cluver LD. Food should not be forgotten: impacts of combined cash transfer receipt and food security on child education and cognition in South Africa and Malawi. AIDS and Behavior. 2021 Sep;25(9):2886-97.
30. Gomez, J. T. "Rainwater-Smart Agriculture in Arid and Semi-Arid Areas: Fostering the Use of Rainwater for Food Security, Poverty Alleviation, Landscape Restoration and Climate Resilience." (2018).
31. Timu, A.G., Gustafson, C.R. and Mieno, T., 2023. The gendered impacts of index-insurance on food-consumption: Evidence from southern Ethiopia. Climate Services, 30, p.100355.
32. Uduji, J.I., Okolo-Obasi, E.N. and Asongu, S.A., 2021. Analysis of farmers’ food price volatility and Nigeria’s growth enhancement support scheme. African Journal of Science, Technology, Innovation and Development, 13(4), pp.463-478.
33. Vilakazi, Thando S. "The causes of high intra-regional road freight rates for food and commodities in Southern Africa." Development Southern Africa 35, no. 3 (2018): 388-403.
34. Winowiecki, L.A., Bourne, M., Magaju, C., Neely, C., Massawe, B., Masikati, P., Vågen, T.G., Musili, F., Nabi, M., Nguyo, A. and Seid, H., 2021. Bringing evidence to bear for negotiating tradeoffs in sustainable agricultural intensification using a structured stakeholder engagement process. International Journal of Agricultural Sustainability, 19(5-6), pp.474-496.
